# Reducing spatial heterogeneity in coverage improves the effectiveness of dog vaccination against rabies

**DOI:** 10.1101/2024.10.03.616420

**Published:** 2024-10-12

**Authors:** Elaine A Ferguson, Ahmed Lugelo, Anna Czupryna, Danni Anderson, Felix Lankester, Lwitiko Sikana, Jonathan Dushoff, Katie Hampson

**Affiliations:** 1 Boyd Orr Centre for Population and Ecosystem Health, School of Biodiversity, One Health & Veterinary Medicine, College of Medical, Veterinary & Life Sciences, University of Glasgow, Glasgow, UK; 2 Environmental Health and Ecological Sciences Department, Ifakara Health Institute, Ifakara, Tanzania; 3 Global Animal Health Tanzania, Arusha, Tanzania; 4 Paul G. Allen School for Global Health, Washington State University, Pullman, Washington, USA; 5 Department of Biology, McMaster University, Hamilton, Ontario, Canada

## Abstract

Vaccination programs are the mainstay of control for many infectious diseases. Heterogeneous coverage is hypothesised to reduce vaccination effectiveness, but this impact has not been quantified in real systems. We address this gap using fine-scale data from two decades of rabies contact tracing and dog vaccination campaigns in Serengeti district, Tanzania. Using generalised linear mixed models, we find that current local (village-level) dog rabies incidence decreases with increasing recent local vaccination coverage. However, current local incidence is most dependent on recent incidence, both locally and in the wider district, consistent with high population connectivity. Removing the masking effects of prior non-local incidence shows that, for the same average prior vaccination coverage beyond the focal village, more spatial variation increases local incidence. These effects led to outbreaks following years when vaccination campaigns missed many villages, whereas when heterogeneity in coverage was reduced, incidence declined to low levels (<0.4 cases/1,000 dogs annually and no human deaths), such that short vaccination lapses thereafter did not lead to resurgence. We inferred ongoing rabies incursions into the district, suggesting regional connectivity as an important source of residual transmission. Overall, we provide an empirical demonstration of how the same average vaccination coverage can lead to differing outcomes based on its spatial distribution, highlighting the importance of fine-scale monitoring in managing vaccination programs.

## Introduction

Vaccination of domestic dogs to interrupt rabies transmission is a quintessential One Health intervention that mitigates risks of human infection from this fatal zoonotic disease [1,2]. While the impacts of vaccinating human populations against childhood diseases have been studied extensively, vaccination of animal populations has not been examined in the same detail, and impacts may differ for a number of reasons. Firstly, demographic turnover and therefore waning of vaccine-induced immunity is likely to be faster in many animal populations than in humans [3–5]; secondly, with the exception of species that undertake long-range migrations [6], animal movement is often more local, perhaps curtailing the geographic spread of disease [7]; and thirdly, vaccinating animals is potentially more difficult, with logistical challenges resulting in lower and/or more heterogeneous coverage [8–11]. The implications of these differences are important to understand for rabies, which kills tens of thousands of people every year and causes billions of dollars of economic losses [12]. Moreover, understanding how vaccination coverage impacts transmission in animal populations could have far-reaching implications for a range of zoonoses and for diseases that threaten food security and endangered wildlife.

Rabies is a fatal viral disease of mammals, with dog-mediated rabies being the cause of >99% of human rabies deaths [13]. Post-exposure vaccines are effective in preventing infection onset, but are costly for both the health sector and for bite victims, and lack of access to these emergency vaccines has tragic consequences [14–16]. However, rabies can be tackled at source through mass dog vaccination. When sufficient coverage is reached, dog vaccination can interrupt transmission and even eliminate rabies, with economic benefits from the reduced need for post-exposure vaccines [17–19]. Dog vaccination is therefore the cornerstone of the global strategic plan, ‘Zero by 30’, to reach zero human deaths from dog-mediated rabies by 2030 [20].

Rabies control by mass dog vaccination is not a new concept. Dog vaccination was integral to the elimination of rabies from Japan in 1957, and is still maintained as a safeguard against reintroductions [21]. Mass dog vaccination was also central to reducing dog-mediated rabies by >95% between 1980 and 2010 in Latin America and the Caribbean [22], with Mexico becoming the first country validated by WHO as free from dog-mediated human rabies since the ‘Zero by 30’ initiative began [23]. Despite these huge strides in the Americas, mass dog vaccination has not been implemented at scale across Africa and Asia. Pilot vaccination projects have reduced local rabies incidence [24–27], dispelled doubts about the accessibility of free-roaming dogs for vaccination, elucidated their role as the reservoir population [28], and provided valuable lessons for improving vaccination campaign design [29–31]. However, piecemeal vaccination efforts are not often maintained or rolled out nationally due to low prioritisation of this neglected disease [32]. Lack of consistent, large-scale vaccination may have negative consequences for the long-term impacts of local efforts. Areas where rabies has been eliminated, but where dog vaccination has lapsed, are vulnerable to reintroductions, as observed in many settings [26,33,34]. Better understanding of incursion risks would inform, for example, how wide vaccination barriers must be to prevent introductions from establishing. Further knowledge of how incursions, local dog movements, and vaccination efforts interact to drive disease dynamics would also provide valuable guidance to rabies-endemic countries as they develop strategic plans to reach the ‘Zero by 30’ goal.

Assessments of mass dog vaccination campaigns suggest that coverage can be highly heterogeneous [29,35,36], often failing to reach the recommended 70% target [25,37]. Reasons for these outcomes include lack of resources and insufficient attention to barriers affecting participation [9,10,35]. This is concerning, as simulation-based models indicate that patches of low coverage could jeopardise rabies control more widely [36,38]. However, the impacts of heterogeneous coverage have rarely, if ever, been quantified using incidence data from a real system, rabies or otherwise. Fine-scale heterogeneities in vaccination coverage may be masked by data aggregated to administrative scales. Such aggregation has been a feature of most empirical studies reporting impacts of rabies vaccination [25,26,39,40] and limits our understanding of how incidence is driven by influences at both local and wider scales. Fine-scale data could allow quantification of these impacts, revealing the consequences of coverage heterogeneities and the extent of epidemiological connectivity.

**Figure 1.**
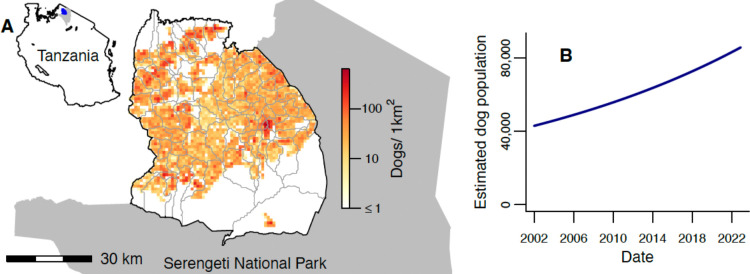
Study area and dog population. A) Serengeti District bordered by Serengeti National Park (grey shading). Grey lines show village borders within the district. The spatial distribution of the estimated dog population at the end of 2022 is indicated by the colour scale (on a log scale). The inset indicates the location of Serengeti District (blue) and Serengeti National Park (grey) within Tanzania. B) Estimated total dog population in Serengeti District from 2002–2022.

Here we examine dog rabies and human rabies exposures and deaths in the Serengeti district of northern Tanzania, where dog vaccination has been ongoing since 2003, alongside contact tracing to track rabies transmission. The district, which had a human population of approximately 340,000 in 2022 [41], is bordered both by other districts where rabies circulates endemically, and by Serengeti National Park where domestic dogs are not permitted (Fig. 1A). Using contact tracing and vaccination campaign data at fine spatiotemporal scales to inform statistical models, we decipher the cross-scale drivers of viral circulation and draw conclusions on what is necessary to eliminate rabies from an animal reservoir in a connected landscape.

## Results

### Heterogeneous vaccination coverage

Serengeti’s human population grew from an estimated 175,017 to 345,587 between January 2002 and December 2022; a growth rate of 3.2% per annum. The dog population, estimated from human:dog ratios, also approximately doubled, increasing from 42,931 to 85,672 (Fig. 1B). Estimated dog densities in December 2022 ranged from 0–625 dogs/km^2^ across a 1km^2^ grid (Fig. 1A, mean of 21 dogs/km^2^ or 37 dogs/km^2^ when considering only cells with a non-zero dog population). Just 5.4% of occupied cells had densities over 100 dogs/km^2^ and <1% exceeded 200 dogs/km^2^.

**Figure 2.**
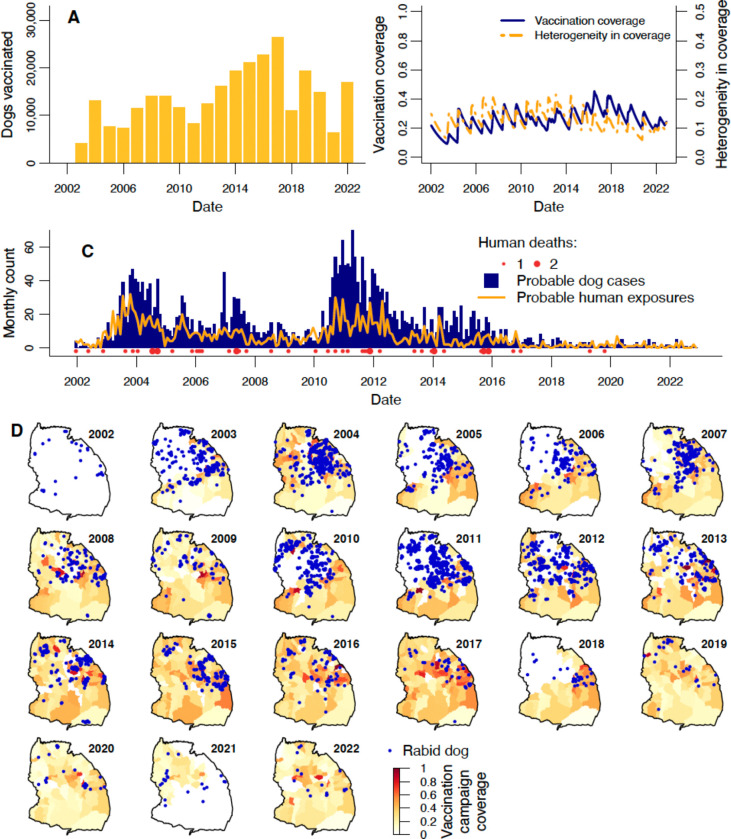
Dog vaccination, dog rabies cases and human rabies exposures and deaths in Serengeti district from 2002–2022. A) Dogs vaccinated annually. B) Monthly estimated dog vaccination coverage, accounting for dog population turnover (blue line), and monthly heterogeneity in vaccination coverage (the population-weighted standard deviation of coverage over all villages in the district; orange line). C) Monthly dog cases, human exposures, and human deaths. D) Village-level coverage (colour scale) achieved in annual vaccination campaigns and locations of dog rabies cases (blue points) each year.

Vaccination campaigns in 2003 reached 4,199 dogs (Fig. 2A) in the district’s southern and eastern villages within 10 kilometres of the Serengeti National Park (~50% of the district’s villages, Fig. 2D) implemented as part of a research project [42,43]. In 2004, in response to a rabies outbreak [44], campaigns were expanded to 90% of villages, with 13,126 dogs vaccinated (Fig. 2A,C-D). Subsequently, campaigns continued in villages bordering the National Park, and less consistently across the rest of the district, dependent on local government resources. Following a resurgence of rabies in 2010–2012, the local government committed to the goal of conducting campaigns annually in all villages throughout the district. Dogs vaccinated steadily increased (Fig. 2A), from 12,532 in 2012 to 26,419 in 2017, i.e. from 21% to 37% of the estimated dog population (supplementary Table S1). Geographic coverage also increased; more villages were included in each successive year, with campaigns in ≥95% of villages throughout 2015–2017. In 2018, vaccination in villages in the northwest of the district lapsed again due to logistical constraints (Fig. 2D). But, in 2019, for the first time, all villages in the district were vaccinated (though the percentage of dogs vaccinated fell below the highs of 2015–2017; supplementary Table S1). In 2021, vaccination did not take place in the southeastern villages for the first time, however as the 2020 campaigns in these villages happened late in the calendar year and the 2022 campaigns were early, the inter-campaign interval was only 17 months for most villages. Hence, the mean proportion of vaccinated dogs in these villages (i.e. considering vaccinations from previous years) did not drop substantially below levels in either 2020 or 2022 (supplementary Fig. S1C).

From 2003 until 2022, the percentage of dogs vaccinated in the district during annual campaigns (hereafter referred to as the “campaign coverage”) ranged from 8–37% (supplementary Table S1), but varied more across villages (Fig. 2D). Of all annual village campaigns over 2003–2022, 23% were missed, while 2.3% reached the recommended target coverage of 70%. The mean village level campaign coverage was 31% (decreasing to 24% when including villages that missed campaigns).

We also estimated the cumulative vaccination coverage as the proportion of the current dog population ever vaccinated, accounting for vaccination timing and dog population turnover; this estimate exceeds campaign coverage, since it includes vaccinated dogs carried over from previous years that were not re-vaccinated. Our estimate of district-level coverage in January 2002 when the study started was 22% based on information from the 2000–2001 vaccination campaign preceding our study [25], with subsequent fluctuation between 9–45% (mean of 26%; Fig. 2B). There was relatively little variation in the magnitude of district-level coverage over the twenty years (Fig. 2B, supplementary Fig. S1A), but coverage between villages was more variable (supplementary Video S1, Fig. S1). Specifically, coverage heterogeneity, calculated as the standard deviation of coverage over all villages, weighted by village dog populations (Fig. 2B), was observed to be higher in 2007 and 2011–2013, but relatively lower from 2019–2022 (Fig. 2B, supplementary Fig. S1B).

### Rabies cases and exposures

A total of 3,362 probable dog rabies cases were identified from January 2002 to December 2022. Annually these varied from just 16 (0.19 cases/1,000 dogs) in 2022 up to 547 (9.4 cases/1,000 dogs) in 2011 and monthly cases ranged from 0 to 70 (Fig. 2C), never exceeding 17 in any given village (supplementary Video S1). Dog cases occurred primarily in the central and northern villages, and were less frequent in the south (Fig. 2D). At least one case was recorded in every village, except for two neighbouring villages in the west of the district. The relatively low dog rabies incidence in 2002 (Fig. 2C) was likely the result of earlier vaccination campaigns that covered most of the district but that lapsed in 2001 [25]. Cases increased over 2003 leading to an outbreak that was largely controlled by the district-wide vaccination campaign in 2004 (Fig. 2D). Further outbreaks (surges with cases exceeding 4/1,000 dogs/year) occurred in 2005 and 2007, during or following years without district-wide vaccination and culminated in the largest outbreak from 2010–2012, which spurred improved vaccination planning. Cases steadily declined from 2012 and remained at low levels of <30 per year (<0.4 cases/1,000 dogs) from 2018 onwards despite lapses in vaccination campaigns in 2018 and 2021 (Fig. 2).

Human rabies exposures (via a bite/scratch from any species) roughly tracked dog rabies, ranging from 0 to 32 exposures per month and peaking in late 2003 (Fig. 2C). Annual exposures ranged from 204 in 2003 (111 exposures/100,000 people) to just 11 in 2021 and 2022 (3 exposures/100,000 people), having declined and remained low (≤21 per year) since 2017. Overall, 48 human rabies deaths were identified (Fig. 2C, supplementary Video S1), with a peak of seven in 2011 (annual incidence of 3 deaths/100,000). Deaths were recorded every year until 2018. Two deaths were recorded again in 2019 despite low numbers of exposures and dog cases, but there were no deaths thereafter. Of the 48 deaths, none completed a full course of post-exposure vaccinations: 3 individuals received one vaccination, 4 received two, and the remaining 41 received none.

### Drivers of disease incidence

**Figure 3:**
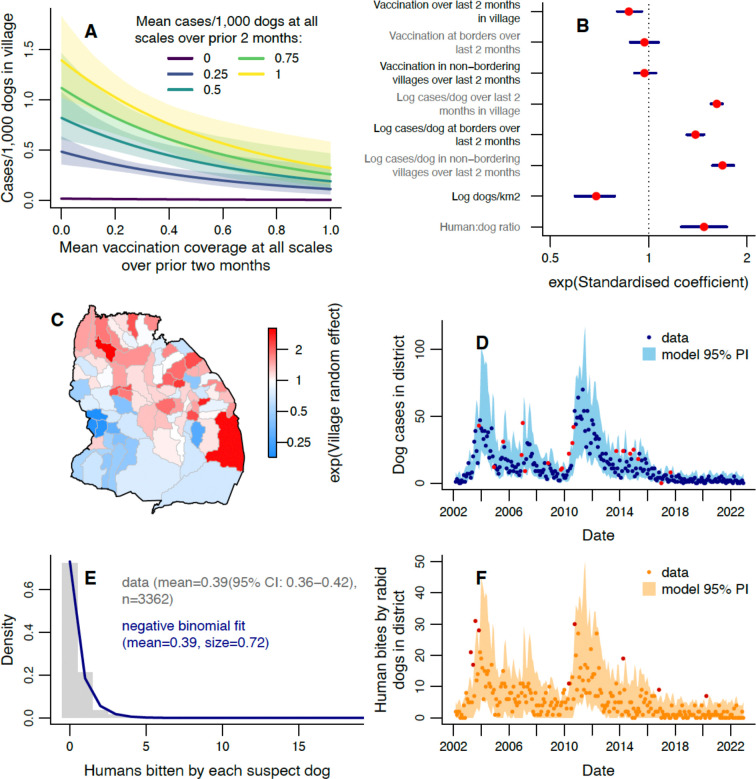
Modelling monthly dog rabies cases in villages. A) Expected cases/1,000 dogs (number of dog cases normalised by dog population) in a village this month for different mean vaccination coverages and mean cases/dog in the prior 2 months. Prior cases/dog values were chosen to represent the range observed at district level and shaded areas show 95% credible intervals (CrIs), with predictions obtained using average values of unspecified explanatory variables. B) Exponentiated standardised values of the coefficients estimated for each explanatory variable, with 95% CrIs. See supplementary Table S2 for tabulated parameter values. C) Exponentiated random effect values for each village. D) Comparison of observed monthly dog cases (points) with the 95% prediction interval from the fitted model. E) Histogram of human rabies exposures by each rabid dog from contact tracing data (grey bars), with fitted negative binomial distribution (blue line). F) Human rabies exposures each month (points), with 95% prediction interval from the dog case predictions in E and the fitted distribution of exposures per dog from E. Data points in red (D, F) fall outside the 95% prediction interval (PI).

To understand the drivers of these infection patterns we used a negative binomial Generalised Linear Mixed Model (GLMM). We modelled current local incidence (cases/dog, i.e. cases in a village, with the logged village dog population as an offset) in response to prior estimated vaccination coverage and prior incidence (mean cases/dog), both averaged over the previous two months (Fig. 2B). Prior coverage and incidence were incorporated at three spatial scales: 1) the focal village; 2) bordering villages (median of 5 villages); and 3) non-bordering villages in the district (across ≥80 villages). Current local incidence decreased with increasing local prior vaccination coverage (Fig. 3C, supplementary Table S2). Specifically, a 10% coverage increase in the focal village was associated with a local incidence decrease of 8.3% (95% credible interval (CrI): 3.2–13.3%), and a 45% coverage increase (the maximum district-wide coverage reached, Fig. 2B) was associated with a local incidence decrease of 32.4% (95% CrI: 13.8–47.5%).

We did not find clear evidence that increased prior vaccination at wider scales (in bordering or non-bordering villages) reduced local incidence (Fig. 3B). However, on fitting a model that excluded prior incidence, an effect of bordering areas became detectable (supplementary Fig. S2B & Table S2). This result likely arises because prior incidence is partly driven by prior coverage, absorbing some of the vaccination effect. The poorer model fit (supplementary Fig. S2D versus 3D) indicates that prior incidence provides explanatory information beyond that shared with prior vaccination. Effects of vaccination beyond the focal village could result from dog owners accessing vaccination campaigns in neighbouring villages, and from reduced incidence in other vaccinated villages within the district lowering the risk of rabid dogs roaming into the focal village.

Increased prior incidence across all three scales was associated with increased local incidence (Fig. 3A-B) and had a greater impact than prior coverage (Fig. 3B), based on the standardised coefficients (found by transforming all explanatory variables to have a variance of one). Logging the prior incidence explanatory variables improved the fit (based on WAIC), as this allows the rate of increase in current local incidence to diminish with increasing prior incidence, possibly accounting for susceptible depletion, or local responses to outbreaks. Prior incidence in bordering villages had less impact on current local incidence than prior incidence in non-bordering villages (Fig. 3B). Doubling mean cases/dog in the focal village over the prior two months led to a 17.3% (95% CrI: 15.7–18.9%) increase in cases/dog, while doubling at all three scales gave a 70.5% (95% CrI: 64.3–76.8%) increase.

Increased dog density in populated areas of a village was associated with reduced cases/dog (Fig. 3B); doubling dog density resulted in a 38.0% (95% CrI: 26.3–48.2%) decrease in expected local incidence. Conversely, local incidence increased with the village’s human:dog ratio (Fig. 3B); increasing the human:dog ratio by one leads to a 39.1% (95% CrI: 20.9–58.4%) increase in incidence. The reasons for these impacts warrant investigation; potentially settings with higher human:dog ratios have less awareness of dog behaviour, leading to rabies circulating with less intervention, while higher dog densities mean more cases are observed at the same incidence, leading to greater visibility and faster intervention. Random effects indicate that villages in the northeast of the district had higher than average incidence (up to 3.2 times; Fig. 3C) while in the southwest, incidence falls to 0.18 times the average in the extreme.

**Figure 4:**
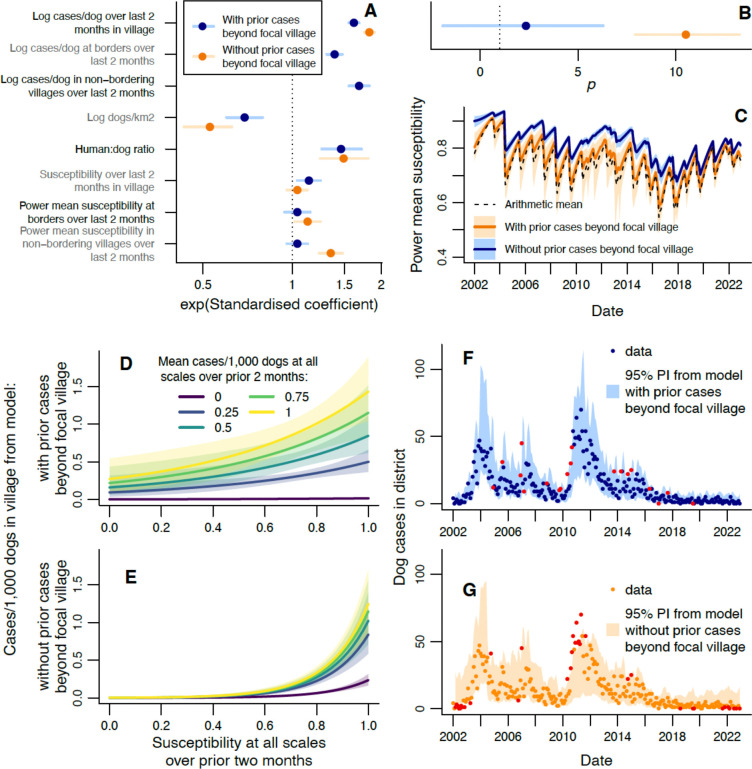
Using power mean susceptibility to model impacts of heterogeneity in vaccination on rabies incidence at the village level. A) Exponentiated standardised estimated coefficients for explanatory variables, with 95% CrIs. B) Estimated power p used to calculate power mean susceptibilities. A-B show estimates from models with (blue) and without (orange) effects of prior incidence beyond the focal village. See Table S2 for tabulated parameter values. C) Monthly arithmetic mean susceptibility over all villages in the district, compared with power mean susceptibility (mean and 95% CrI) from fitted values of p from models with and without prior incidence beyond the focal village. D-E) Expected cases/1,000 dogs in a village from models with (D) and without (E) effects of prior incidence beyond the village. Predictions are shown for different mean susceptibilities (assuming homogeneous vaccination, i.e. power mean susceptibilities beyond the village equal susceptibility in the village) and mean cases/dog in the prior 2 months. Prior cases/dog values represent the observed district-level range and shaded areas show 95% CrIs, Predictions were obtained using average values of unspecified explanatory variables. F-G) Comparison of observed monthly dog cases (points) with the 95% prediction interval from the fitted model with (F) or without (G) prior incidence beyond the village. Data points in red fall outside the 95% prediction interval (PI).

To explore the impact of vaccination heterogeneity, we also fitted a model where prior vaccination coverages in bordering and non-bordering villages were replaced with prior power means of susceptibility (1 - vaccination coverage) at the same scales. For this model, it was necessary to fit the power p used in calculating the power means (equation 13). If p=1, the power mean equals the arithmetic mean, and we revert to the original model, where heterogeneity does not affect vaccination impacts on incidence. If p>1, then homogeneous vaccination would still give a power mean equal to the arithmetic mean, but heterogeneous coverage would result in villages with higher susceptibilities having a greater weighting on the power mean, which rises above the arithmetic mean; i.e. for the same total number of unvaccinated dogs, the effective susceptibility is higher when these dogs are distributed unevenly within the district. If p<1, we have the opposite scenario, where heterogeneity reduces effective susceptibility. On fitting this model, we initially fail to detect an impact of effective susceptibility in bordering and non-bordering villages (Fig. 4A-B). However, on refitting without the effects of prior incidence in bordering and non-bordering villages (on the basis that the heterogeneous vaccination landscape may be directly responsible for incidence in these areas, so including these incidence effects may mask vaccination impacts), we find that incidence increases with effective susceptibility at both of these wider scales. We estimate p to be substantially larger than one (Fig. 4A-B), and beyond what we *a priori* expected to be the feasible range (Fig. S3). The fit of this model is poorer than that including prior incidence beyond the village (Fig. 4F-G), likely because other contributing factors (e.g. incursions and spatial aggregation of low-coverage villages) are no longer being indirectly accounted for. We also find that the effect of local susceptibility on local incidence is no longer clearly detectable, possibly a consequence of limited data. The increase in effective district-level susceptibility caused by heterogeneity (Fig. 4C, supplementary Fig. S4) follows a similar pattern to the standard deviation in village coverage (Fig. 2B, supplementary Fig. S1B), having peaks in 2007 and 2013, and being lower from 2019. This model predicts, for example, that if half the bordering and non-bordering villages had prior coverage of 30%, with the other half having 50% then the focal village incidence will be 1.4 (95% CrI: 1.3–1.6) times that had the prior coverage beyond the village been homogeneous at 40%. If we further increase heterogeneity by assuming half the villages had prior 20% coverage and the other half 60%, then local incidence is expected to be 2.6 (95% CrI: 1.9–3.4) times the homogeneous scenario.

We also explored different village/district and monthly/annual model combinations. When modelling monthly district-level incidence, prior incidence remained a strong predictor, but we found no clear impact of prior district-level vaccination or power mean susceptibility (even on removal of prior incidence) (Figs S5–6, Table S3). For annual village-level models we consistently identified an effect of prior vaccination in the focal village, but rarely of vaccination beyond the focal village (Fig. S7, Table S4). In annual district-level models we consistently did not find a clear impact of prior vaccination coverage (Fig. S8, Table S5). Reduced ability to detect effects in these models likely results from the much reduced dataset (20 annual district-level data points), demonstrating the limitations of using coarsely aggregated data.

Individual rabid dogs bit between 0–18 people (mean=0.39, Fig. 3E), with 72% not biting anyone, and 21% biting just one person. Using a fitted negative binomial distribution (mean=0.39 (±0.013, standard error); size=0.72 (±0.066); Fig. 3E) to simulate human exposures from monthly village-level GLMM simulations of rabid dogs (Fig. 3D) provided a good match to observed exposures (Fig. 3F). Of all human exposures, 87.5% were due to domestic dogs (Fig. 2C) and exposures from other species followed a similar pattern as those by dogs (supplementary Fig. S9). Of non-dog-mediated human exposures where the biting species was known, 47.9% were by domestic cats, 11.6% by livestock, 2.1% by humans, and the remaining 38.4% by various wildlife species (with half of these being by jackals).

**Figure 5.**
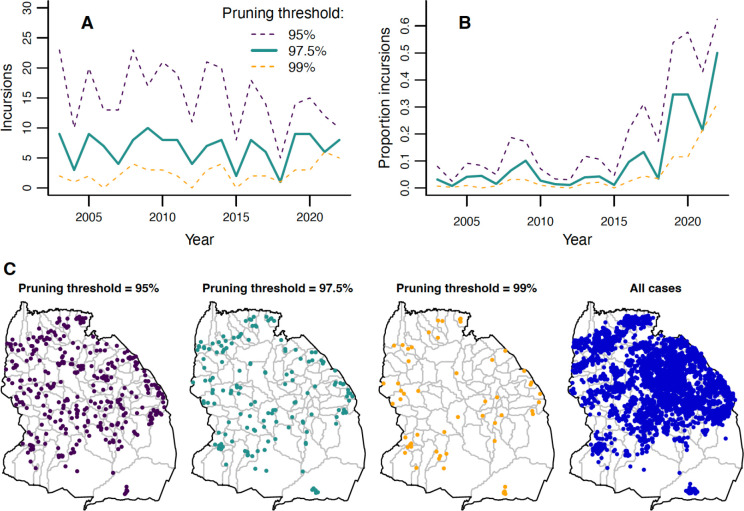
Estimated incursions in Serengeti District over time and space. A) Annual numbers of incursions inferred from transmission trees with three different pruning thresholds. B) Annual proportion of total rabies cases in carnivores that are inferred to be incursions. C) Locations of inferred incursions under the three pruning thresholds. Locations of all carnivore cases are shown for comparison.

By reconstructing transmission trees, we identified probable incursions as cases without a plausible parent case within the 97.5th percentile of the distance kernel and serial interval distributions (supplementary Fig. S10 and Table S6), i.e. the 97.5% pruning threshold. Numbers of incursions remained relatively constant from 2003–2022 (Fig. 5A, mean of 7 annual incursions). In contrast, the *proportion* of cases identified as incursions sharply increased (Fig. 5B), from 3% pre-2018 to 26% post-2018, peaking at 50% in 2022. This result suggests increased relative importance of incursions versus local transmission in maintaining viral circulation in later years. Incursion locations were more focused along the district’s edges than cases in general (Fig. 5C). Adjusting the pruning threshold to 95% or 99% impacted the number of inferred incursions, but led to qualitatively similar temporal and spatial patterns (Fig. 5).

## Discussion

We identify key drivers of rabies transmission from twenty years of fine-scale data on dog vaccination and from contact tracing. Under endemic rabies circulation, we find that outbreaks occur following years when clusters of villages remain unvaccinated. However, when district-wide vaccination is routine and spatial heterogeneity in coverage reduced, incidence declines to low levels (<0.4 cases per 1,000 dogs annually), attributable largely to short chains of transmission following incursions into the district. Recent rabies incidence locally (at the scale of the focal village) and across the district was the main driver of current local incidence, but was modulated by recent local vaccination coverage. When the masking effect of non-local rabies incidence was removed, we also identified impacts of non-local vaccination coverage and heterogeneity in this coverage. The role of prior incidence was highlighted in later years after vaccination had largely interrupted endemic transmission, when rabies did not re-surge despite late and incomplete vaccination campaigns. Reducing dog rabies cases had dramatic public health benefits, with corresponding reductions in human rabies exposures and deaths. However, frequent incursions suggest that benefits may be short-lived if dog vaccination lapses for extended periods.

Our analysis reveals how fine-scale variation in vaccination coverage drives rabies dynamics, with models aggregating coverage to district level failing to show impacts on rabies incidence. Our empirical findings support simulation-based work arguing that gaps in coverage are detrimental for rabies control [36,38]. Pockets of low coverage are recognized as a driver of measles outbreaks [45–48], but remain underexplored for other diseases, despite examples of vaccination heterogeneity in systems from cholera [49] to COVID-19 [50]. The impact of this heterogeneity on disease incidence has rarely, if ever, been quantified in real systems, likely due to a lack of fine-scale data. Our work addresses this gap, providing evidence that the same level of vaccination coverage can have substantially different impacts, based on the spatial distribution of that coverage. Future studies may be aided by models that predict fine-scale vaccination coverage from coarser available data, e.g. sparse household surveys [51] or aggregated areal data [48]; though while these models may work well for routine childhood vaccinations, their transferability to the stochasticity arising from single-day village-level campaigns employed for rabies is unknown. The potential impact of clusters of low-coverage villages also requires further exploration. Nonetheless, striking district-level impacts of coverage were found during large-scale rabies vaccination across 13 contiguous districts in south-east Tanzania, without accounting for heterogeneity [39]. These contrasting results were likely due to our focus on a single district without wider-scale vaccination to reduce spread between districts. Our focus on a single, possibly idiosyncratic, time series may also be why the posterior for the parameter p that governs the heterogeneity effect lay outside the region that we *a priori* believed feasible; data from other areas could help refine estimates in future.

We find that vaccination controlled rabies despite not reaching the recommended 70% coverage target [25,37]. Some dog vaccination campaigns have faced similar difficulties in achieving high coverage [8,52], while others (even in Serengeti) have reported more success [25,53–55]. The low coverages we report may reflect our estimation methods [56]. We used vaccination records, with the dog population denominator derived from censuses and human:dog ratios. Methods like post-vaccination transects or household surveys may overestimate coverage if they rely on owner recall or do not cover unvaccinated sub-populations, resulting in bias towards more accessible areas and more visible (often adult) dogs [56,57]. Assuming constant human:dog ratios may have reduced the accuracy of our coverage estimates, while the relatively dense dog population and low human:dog ratio (versus [58] and [59]) may have contributed to the low coverage attained. Nonetheless, district coverage only fell below 20% – the critical threshold estimated to push the reproductive number below one [44] – on <5% of months post-2007 (Fig. 2B). So, while higher coverage would have achieved better (and faster) results, rabies was controlled regardless. Similarly, modelling suggests vaccination coverages of ≤40% could eliminate rabies [38] and protect against catastrophic declines in endangered canids [60]. We therefore conclude that, while high coverage should be the target for rapidly controlling disease, policymakers should not be deterred from introducing dog vaccination when achieving 70% coverage is challenging; if persistent spatial gaps are minimal, sub-optimal coverage still has major benefits due to rabies’ low transmissibility [44] relative to human diseases that require higher levels of vaccination [61]. However, fast demographic rates still necessitate a large number of dogs be vaccinated annually to maintain coverage, in comparison to childhood vaccination programmes where higher coverage is reached through targeting a narrower demographic [62].

Impacts of prior rabies incidence at wider spatial scales on local incidence, which are driven in part by vaccination coverage at those scales, indicate high epidemiological connectivity. Therefore, high coverage and resulting low incidence elsewhere mitigate local coverage gaps by reducing rabid dog movement into these areas. In contrast, high coverage in a small area has only limited impact if the rest of the population is poorly vaccinated, explaining why reactive vaccination is ineffective when targeted by weak surveillance [38,63]. This interplay between dog vaccination and movement may have complex impacts on incidence that are also influenced by population configuration. Studies from Serengeti district [64] and Bali, Indonesia [65] identified impacts of prior incidence in nearby areas on rabies occurrence. Unlike those studies, we did not detect decreased impacts with distance from the focal village, possibly because we studied only the collective impacts of non-bordering versus bordering villages (80+ vs ~5); evaluating individual village impacts would be more likely to reveal distance-related effects.

We inferred an average of 7 incursions annually, with incursions more frequent near district borders. Incursions in the district centre likely result from human-mediated transport, which is an increasingly recognized problem [33,36,38,66,67]. Our inference methods have limitations; local transmission could be misidentified as incursions due to unobserved transmission or unusually long incubation periods or dog movements, while real incursions could go undetected within existing foci. We expect less misidentification at low incidence, suggesting inferred incursions should have increased post-2018. However, no increase was observed, possibly because real incursions decreased from the indirect effects of vaccination on circulation in neighbouring districts and/or the direct impact of vaccination in those districts from 2020 [30,68]. Misidentification may contribute to fewer inferred incursions in the district centre, where incidence was generally higher. Simulation studies could validate the performance of incursion assignment, and incorporating viral genomes may improve accuracy [33]. Regardless, the proportion of cases identified as incursions is far higher in later years (>20% of cases since 2019, reaching as high as 50% in 2022). This encouraging indication that local transmission has been interrupted, also serves as a warning that, no matter how well district-level control measures are implemented, elimination will require expanded vaccination. The example of Latin America shows how scaled up vaccination has largely eliminated dog-mediated rabies, with a contracting set of foci remaining in only the most challenging settings [22,40]. In Tanzania, rabies vaccination in Serengeti district began to extend across the surrounding Mara region in late 2020 [30,68]. Estimated incursions did not decline in 2021–2022, but this may change as rabies is controlled in neighbouring districts.

We conclude that dog vaccination was only effective in interrupting endemic circulation when implemented consistently, underlining the importance of monitoring fine-scale coverage to promptly identify and address gaps, which we found to be highly detrimental for control. Rabies was nonetheless controlled with relatively low coverage; a promising result given hard-to-reach populations with fast demographic rates. High epidemiological connectivity within the district, and frequent incursions from outside, interact with heterogeneous vaccination in complex ways that should be considered in programme design. Continued introductions from unvaccinated areas can set back local control efforts. It is now vital that dog vaccination efforts be scaled up to ensure that impacts are sustained, to maximise their benefit to all, and to achieve the ‘Zero by 30’ goal.

## Materials and Methods

### Dog vaccination

We focus on the epidemiological dynamics of rabies in Serengeti District, northwest Tanzania from January 2002 to December 2022 during which time we undertook contact tracing and monitored mass dog vaccination. Prior to this, between October 1996 and February 2001, four vaccination campaigns were carried out in Serengeti District [25]. These campaigns covered all the villages in the district as it was then, but we note that the district has since increased in area, with new villages incorporated that were previously part of the adjacent district, Musoma, and that were not covered in those earlier campaigns (compare Serengeti District maps in Fig.1 to in Cleaveland et al. [25]). Following the last of these four campaigns, household surveys estimated 73.7% coverage in the district. Vaccination campaigns did not restart in Serengeti until 2003, when the Serengeti Health Initiative began annual campaigns in the villages in the East and South, aiming to protect people and their animals and to prevent rabies spilling over into vulnerable carnivore populations in Serengeti National Park [42]. In 2004, vaccination was expanded to more villages with support from the District Veterinary Office.

Dog vaccination typically takes place annually using a central point strategy where dogs are brought to a central location, such as a village centre, church, or school in each village. A vehicle equipped with a loudspeaker is used to advertise vaccination the day before the campaign. Posters advertising the vaccination day are hung at busy locations within each village, such as village headquarters office, dispensaries, schools, churches and mosques. On vaccination days, dogs are registered, recording owner name, dog name, age, and sex. Rabies vaccination is offered free of charge using the Nobivac Rabies vaccine (MSD Animal Health, Boxmeer, The Netherlands). Dog owners receive a vaccination certificate for each vaccinated dog.

Dogs are occasionally vaccinated outside of central point campaigns in response to outbreaks. Since late 2020, villages in the northwest of the district have been part of a large-scale vaccination trial across Mara Region. A subset of those villages are following a continuous strategy, where vaccinations happen year-round [30] while the remaining villages continue annual central-point campaigns. Date, village and number of dogs vaccinated are available for all described vaccination events throughout the study period.

### Rabies incidence and exposures

Contact tracing was carried out following the methods outlined in Hampson et al. [44] to generate data on 3,973 probable animal rabies cases – of which 3,362 were domestic dogs, 368 were livestock, 159 were wildlife, 81 were domestic cats, and three did not include a record of species – and 1,612 probable human rabies exposures in the years 2002–2022. It has previously been estimated that these methods identify 83–95% of carnivore cases in the district [69]. Each animal case and human exposure was georeferenced and time-stamped, with the identity of the biting animal recorded where possible.

### Dog population estimation

Human population counts were available at the village level from the 2012 government census [70], and at ward-level for the 2002 and 2022 censuses [41]. Village-level estimates for 2002 and 2022 were obtained by assuming that the population in each ward was divided between the villages in that ward in the same proportions as in 2012. For each village v, we then estimated the human population H for each month m in 2000–2022 to be:

(1) $$H_{v,m}=\alpha_{v}e^{r_{v}m}$$

where parameters $a_{v}$ and $r_{v}$ were estimated for each village using the three census populations by non-linear least squares.

A georeferenced household census of humans and dogs in every village in Serengeti District was completed between 2008 and 2016 [56], allowing estimation of village-specific human:dog ratios $R_{v}$. The dog population in every village each month was estimated as:

(2) $$D_{v,m}=\frac{H_{v,m}}{R_{v}}$$

We also mapped the dog population to a grid of 1km^2^ cells by first assigning each cell to a village within Serengeti district. For each village, the dog population in each month $\left(D_{v,m}\right)$ was then distributed among cells of that village in proportion to the number of dogs in those cells at the time of the georeferenced census.

### Vaccination coverage

We calculated the numbers of dogs vaccinated in each village in each month in 2000–2022, $V_{v,m}$. To allow estimation of existing levels of vaccination at the time contact tracing began in 2002, we included information about the final vaccination campaign in the district prior to 2002, which was completed in 2000–2001 [25]. Monthly village-level data were not available for this campaign, so we assumed that the 73.7% of dogs estimated to have been vaccinated by post-vaccination household surveys (completed within two days of the vaccination campaign in each village) were distributed evenly across the campaign months (May 2000 to February 2001) in all villages that were part of the district at that time.

Village campaign coverages each year y (Fig. 2D) were calculated as:

(3) $$P_{v,y}=b\left(\sum_{\mu }\left(\frac{V_{v,\mu }}{D_{v,\mu }}\right)\right)$$

where μ are the months of y. To prevent estimates of $P_{v,y}>1$ (e.g. due to dog populations being underestimated or dogs being taken for vaccination outside their home village), we use the bounding function b(x):

(4) b(x)=x1+xa1a

where we set a=6. This means that values of x<0.7 experience little change, while x=1.0 would be reduced to a more realistic 0.89 (Fig. S11).

Annual district campaign coverages (supplementary Table S1) were calculated as:

(5) $$P_{y}=100\left(\sum_{\mu }\left(\frac{\sum_{v=1}^{88}V_{v,\mu }}{\sum_{v=1}^{88}D_{v,\mu }}\right)\right)$$

When estimating monthly cumulative vaccination coverages (i.e. the proportion of dogs in the current month that have been vaccinated at least once, not necessarily in the current year; Fig. 2B, supplementary Video S1), we assume that numbers of vaccinated dogs decline geometrically each month with ratio:

(6) $$\lambda =\sqrt[{12}]{1-d}$$

where d=0.448 is the proportion of dogs that die in a year [71]. Assuming that all dogs are equally likely to be vaccinated each year, regardless of previous vaccination status, and that the same dog cannot be vaccinated twice in the same year, the number of vaccinated dogs in a village in a given month can be estimated as

(7) $$N_{v,m}=b\left(\left(\lambda N_{v,m\;-\;1}+V_{v,m}\left(1-p_{v,m}\right)\right)/D_{v,m}\right)D_{v,m}$$

where $p_{v,m}$ is the proportion of dogs that are available to be vaccinated this month (i.e. not already vaccinated in the current year) that had already been vaccinated in a previous year:

(8) $$p_{v,m}=\frac{n_{v,m}}{D_{v,m}\;-\;\left(\lambda N_{v,m-1}\;-\;n_{v,m}\right)}$$

and $n_{v,m}$ is the number of vaccinated dogs that were not vaccinated in the current year:

(9) $$n_{v,m}=\left\{\begin{array}{ll} \lambda N_{v,m-1} & \text{if}\;\text{January}\\ \lambda max\left(0,n_{v,m-1}-V_{v,m-1}p_{v,m-1}\right) & \text{if}\;\text{any}\;\text{other}\;\text{month} \end{array}\right.$$

Finally, we obtained vaccination coverage for every village and month (supplementary Video S1) by:

(10) $$C_{v,m}=\frac{N_{v,m}}{D_{v,m}}$$

and vaccination coverage over the district in each month (Fig. 2B) by:

(11) $$C_{m}=\frac{\sum_{v=1}^{88}N_{v,m}}{\sum_{v=1}^{88}D_{v,m}}$$

The level of heterogeneity in coverage over the district each month $H_{m}$ (Fig. 2B) was quantified using the population-weighted standard deviation in village coverage:

(12) $$H_{m}=\sqrt{\sum_{v=1}^{88}\left(\frac{D_{v,m}}{\sum_{v=1}^{88}D_{v,m}}{\left(C_{v,m}-\sum_{v=1}^{88}\left(\frac{D_{v,m}}{\sum_{v=1}^{88}D_{v,m}}C_{v,m}\right)\right)}^{2}\right)}$$

### Modelling the impact of vaccination on rabies incidence

To investigate the impact of vaccination and other variables on rabies incidence, we developed a negative binomial generalised linear mixed model (GLMM), where the response variable was the number of traced probable dog rabies cases in a village in a given month. An offset equal to the log of the estimated dog population $D_{v,m}$ was included, allowing us to model incidence as cases per dog. Village was included as a random effect.

Since >90% of rabies incubation periods recorded from contact tracing were less than two months, we include mean estimated vaccination coverage in the village v over the prior two months $\left(C_{v,m-1}+C_{v,m-2}\right)/2$ as a fixed effect. As we also wanted to explore impacts of conditions in areas outside the focal village on local incidence, we included fixed effects of mean vaccination coverage at two wider scales over the prior two months. The first of these was at the borders of the focal village. We obtained this variable by identifying all villages that shared a border with the focal village and calculating the proportion of the border shared with each of these villages. Bordering vaccination coverage in a given month was then calculated by multiplying these border proportions by vaccination coverages in these bordering villages, and summing over the bordering villages, i.e. the border-weighted arithmetic mean coverage. Vaccination coverage at borders with Serengeti National Park was assumed to be equal to the average of the vaccination coverages in the other bordering villages, and coverage at borders with the rest of Mara District was set to 9% (the coverage estimated in Serengeti District prior to mass vaccination campaigns [25]). Finally, we introduced a fixed effect of the prior population-weighted arithmetic mean vaccination coverage over the dog populations of the rest of the district villages not sharing a border with the focal village.

To explore the impact of heterogeneity in coverage within the district on incidence in focal villages, we also considered a version of this model where the arithmetic mean coverages over bordering and non-bordering villages were replaced by power mean susceptibility (where susceptibility=1-coverage) at these scales. A power mean $M_{p}$ over a sample, is calculated as:

(13) $$M_{p}\left(x_{1},\ldots ,x_{n}\right)={\left(\frac{\sum_{i=1}^{n}w_{i}x_{n}^{p}}{\sum_{i=1}^{n}w_{i}}\right)}^{1/p}$$

where $w_{i}$ are the weights applied to each observation (length of shared border for mean susceptibility of bordering villages, and dog population for mean susceptibility of non-bordering villages). When fitting this model, we estimated the value of p in addition to the two GLMM coefficients for power mean susceptibility in bordering and non-bordering villages. We focus on power means of susceptibility rather than of vaccination coverage to enable calculation of logarithms of the power mean elements (to allow first-order approximation when p is close to zero and to provide stabilisation at large p); vaccination coverages of zero are plausible and occur frequently in the data, while susceptibilities of zero are both implausible in this setting, and corrected by equation (4).

Current incidence of dog rabies was expected to be highly dependent on prior incidence, so we included an effect of mean incidence in the focal village over the prior two months. Prior incidence at the wider spatial scales was included as described for vaccination. Incidence at borders with the rest of Mara Region was assumed to be 0.01/12, as the highest proportion of Serengeti’s dog population infected in any given year was 0.0094, and the rest of Mara Region was largely unvaccinated so presumably had higher incidence. Incidence at the borders with Serengeti National Park was assumed to be equal to the average incidence in the other villages bordering a focal village. We compared a model where the three prior incidence variables were logged with one where they were unlogged, selecting the best based on WAIC.

We incorporated fixed effects of the log of dog density in the village and the human:dog ratio $R_{v}$. When calculating dog density in a village we used the number of 1km^2^ grid cells assigned to the village that were occupied by at least one household during the Serengeti dog census as the denominator. This denominator was chosen to account for the fact that many villages, particularly on the North and South borders (Fig. 1A), have large unoccupied areas that would otherwise bias density estimates.

In addition to the models described above, which use data at relatively fine spatiotemporal scales (village and month), we investigated impacts of vaccination at the remaining three combinations of village/district and monthly/annual scales. At the monthly, district scale, a negative binomial generalised linear model (GLM) was fitted, where the response variable was cases in the district each month, with an offset of the logged dog population in the district. Fixed effects included estimated vaccination coverage (or power mean susceptibility) and rabies incidence in the district averaged over the prior two months, and logged dog density.

The third set of models at the annual, village scale, was fitted as GLMMs with a response of the number of cases in a village in each year, with an offset of the logged mean village dog population that year. Prior vaccination at the three spatial scales (village, borders, rest of district) was incorporated in the form of the campaign coverage $P_{v,y}$ (equation (3), Fig. 1D). We fitted models with fixed effects of: 1. $P_{v,y}$ last year; 2. Mean $P_{v,y}$ over the last two years; 3. Mean $P_{v,y}$ over the last three years; 4. $P_{v,y}$ last year and logged incidence last year. Log dog density and human:dog ratio were also included in all four models, with village as a random effect. All fixed effect combinations are included in supplementary Table S4.

Finally, we fitted a fourth set of models at the annual, district scale that were GLMs with a response of cases in the district each year and an offset of the logged district dog population. The campaign coverage in the district each year $P_{y}$ (equation (5), Table S1) was included in the same four combinations as for $P_{v,y}$ in the annual village-level models. All of these models included log dog density as a fixed effect (see supplementary Table S5 for details).

All models were fitted using Stan [72], via the RStan package [73] for models that included power means of susceptibility to allow estimation of both the power and coefficients and otherwise via the brms package [74]. For all variable coefficients we assumed a normal prior, N(μ=0,σ=100,000). For the size parameter of the negative binomial distribution, we use a gamma prior, G(ɑ=0.01,β=0.01) (the default prior selected for this parameter in brms). An exponential prior, Exp(λ=0.001), was used for the standard deviation of the village random effect in village-level models. To discourage what we believed to be unrealistically large effects of heterogeneous coverage (Fig. S3), we used a normal prior for p centred on the arithmetic mean with a standard deviation of two, N(μ=1,σ=2). For models fitted in brms, we checked model assumptions using simulated residuals via the DHARMa package [75].

### Estimating incursions

To identify cases that likely originated from transmission outside Serengeti District (i.e. incursions), we reconstructed transmission trees using the treerabid package in R [69,76]. For this analysis, we excluded cases in herbivores, since these are unlikely to cause onward transmission, reducing the analysed cases to 3,600. Tree reconstruction required distributions for the serial interval S (the time between onset of symptoms in an offspring case and in its parent case) and dispersal kernel K (the distance between cases and contacts regardless of whether these developed rabies). We calculated serial intervals from 1,156 paired dates of symptoms onset from cases and their recorded rabid biter and Euclidean distances between locations of 6,897 contacts and their recorded biter. For both S and K we fitted gamma, lognormal and Weibull distributions using the fitdistrplus package [77], with the best-fitting distributions - lognormal for S and Weibull for K - chosen based on AIC (supplementary Table S6). While fitting these distributions, censoring was applied for distances as described previously [69]. Distances of <100m where the biter was a dog with known owner, and an accurate home location, were interval censored between 0 and 100m to account for homestead sizes. For unknown dogs and wildlife, where locations were recorded for first observation rather than home location, the true distance to contacts was assumed unknown (right censored), but with a minimum of the recorded value or 100m, whichever was larger, on the basis that biters from <100m away would be recognised. Parameters of the fitted distributions (supplementary Table S6) have changed little from those estimated previously [69], despite an additional seven years of contact tracing. A comparison of the data and best-fitting distributions is shown in supplementary Fig. S10.

The transmission tree reconstruction algorithm [76] was used to probabilistically assign a progenitor to each case i with no known rabid biter (65.75% of all carnivore cases). To be considered a possible progenitor of i, a case j has to have a symptoms onset date preceding that of i, and the serial interval and distance between i and j must be smaller than a selected quantile of the distributions S and K. This quantile, known as the pruning threshold, is chosen to prevent assignment of progenitors that are unlikely to be correct given the separation of cases in space or time. We explored pruning thresholds of 0.95, 0.975, and 0.99; the maximum serial intervals and distances defined by these thresholds are given in supplementary Table S6. The probability of each case j∈{1,…,n} that meets these pruning criteria being randomly selected as the progenitor of i is:

(14) $$p_{ij}=\frac{S_{ij}K_{ij}}{\sum_{k=1}^{n}S_{ik}K_{ik}}$$

where $S_{ij}$ and $K_{ij}$ are the probabilities of the serial interval and distance between i and j based on reference distributions S and K. In cases where n=0, no biter was assigned, with i being assumed to be an incursion. To account for recorded uncertainties in the timing of bites and symptoms onset, 1,000 bootstrapped datasets were generated by drawing dates randomly from a uniform distribution over the recorded uncertainty window, while preserving the correct sequence of events between known parent and offspring cases. We used the best-fitting lognormal distribution for S (supplementary Table S6) and when i was a dog with known owner and accurate location we used the best-fitting Weibull distribution for K. When i was an unknown dog or wildlife, where the recorded location was generally the location i was observed biting, not i’s home location, K was a Weibull distribution fitted to simulations of the Euclidean distance travelled as a result of two draws from the previously fitted Weibull with a uniform random change in direction between them (representing the movement of j to i, followed by the movement of i to its recorded location). For each pruning threshold, we identified probable incursions as those cases identified as an incursion more frequently than they were assigned to any other progenitor within the set of 1,000 bootstrapped trees. Inferred incursions and proportion incursions were notably high in 2002, as an expected artefact of beginning contact tracing, and are thus not considered in the results. Progenitors of some 2002 cases likely occurred in 2001, and thus would have gone undetected. Additionally, detection was likely lower in 2002 as methods were still being honed.

All analyses were carried out using the R statistical computing language, version 4.2.0 [78]. Deidentified data and code files are located in our Github repository: https://github.com/boydorr/Serengeti_vaccination_impacts.

Ethical approval for this research was obtained from the Tanzania Commission for Science and Technology, the Institutional Review Boards of the National Institute for Medical Research in Tanzania and of Ifakara Health Institute, and the Ministry of Regional Administration and Local Government (NIMR/HQ/R.8a/vol.IX/300, NIMR/HQ/R.8a/vol.IX/994, NIMR/HQ/R.8a/vol.IX/2109, NIMR/HQ/ R.8a/vol.IX/2788, and IHI/IRB/No:22-2014).

## Supplementary Material

Supplement 1
