# Supplementary material for: Reducing spatial heterogeneity in coverage improves the effectiveness of dog vaccination campaigns against rabies
Source: bioRxiv. 2025 Jan 16:2024.10.03.616420. Preprint. [Version 3] doi: 10.1101/2024.10.03.616420 (PMC11482771; doi:10.1101/2024.10.03.616420)
Supplement: Supplement 1 [file NIHPP2024.10.03.616420v3-supplement-1.pdf]

bioRxiv preprint doi: https://doi.org/10.1101/2024.10.03.616420; this version posted January 16, 2025. The copyright holder for this preprint (which was not certified by peer review) is the author/funder, who has granted bioRxiv a license to display the preprint in perpetuity. It is made available under aCC-BY 4.0 International license.

Supplementary information

Supplementary Videos

Video S1

Video S1: Monthly spatial distribution of dog cases, human exposures and dog vaccination coverage over the study period. Estimated vaccination coverage at the village-level for each month in 2002-2022 is indicated by the colour scale. Locations of dog cases (blue points), human exposures (purple triangles) and human deaths (large black triangles) each month are indicated.

bioRxiv preprint doi: https://doi.org/10.1101/2024.10.03.616420; this version posted January 16, 2025. The copyright holder for this preprint (which was not certified by peer review) is the author/funder, who has granted bioRxiv a license to display the preprint in perpetuity. It is made available under aCC-BY 4.0 International license.

Supplementary Figures

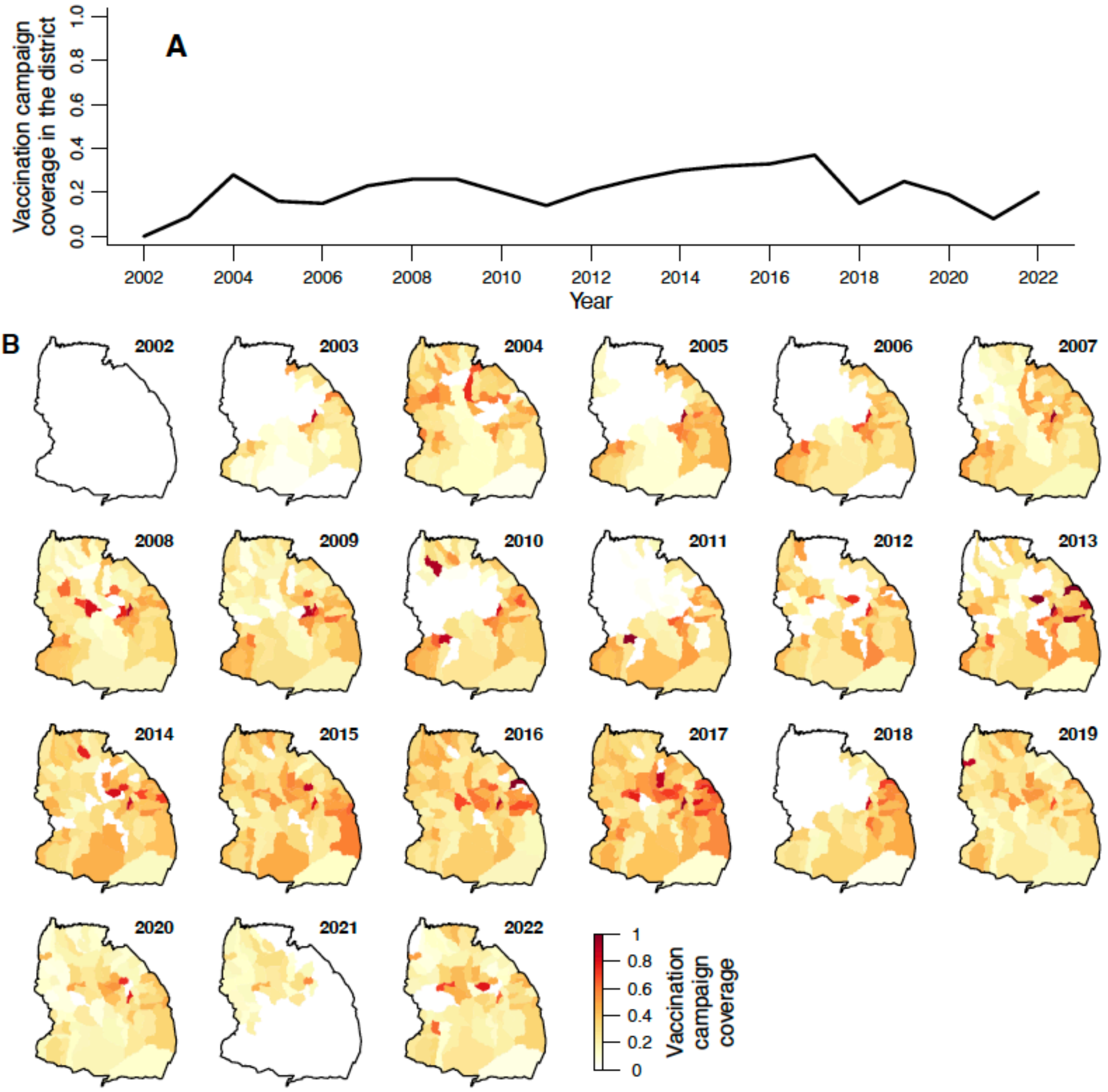

Figure S1. Campaign vaccination coverage each year from 2002-2022 A) at district level and B) at village level.

bioRxiv preprint doi: https://doi.org/10.1101/2024.10.03.616420; this version posted January 16, 2025. The copyright holder for this preprint (which was not certified by peer review) is the author/funder, who has granted bioRxiv a license to display the preprint in perpetuity. It is made available under aCC-BY 4.0 International license.

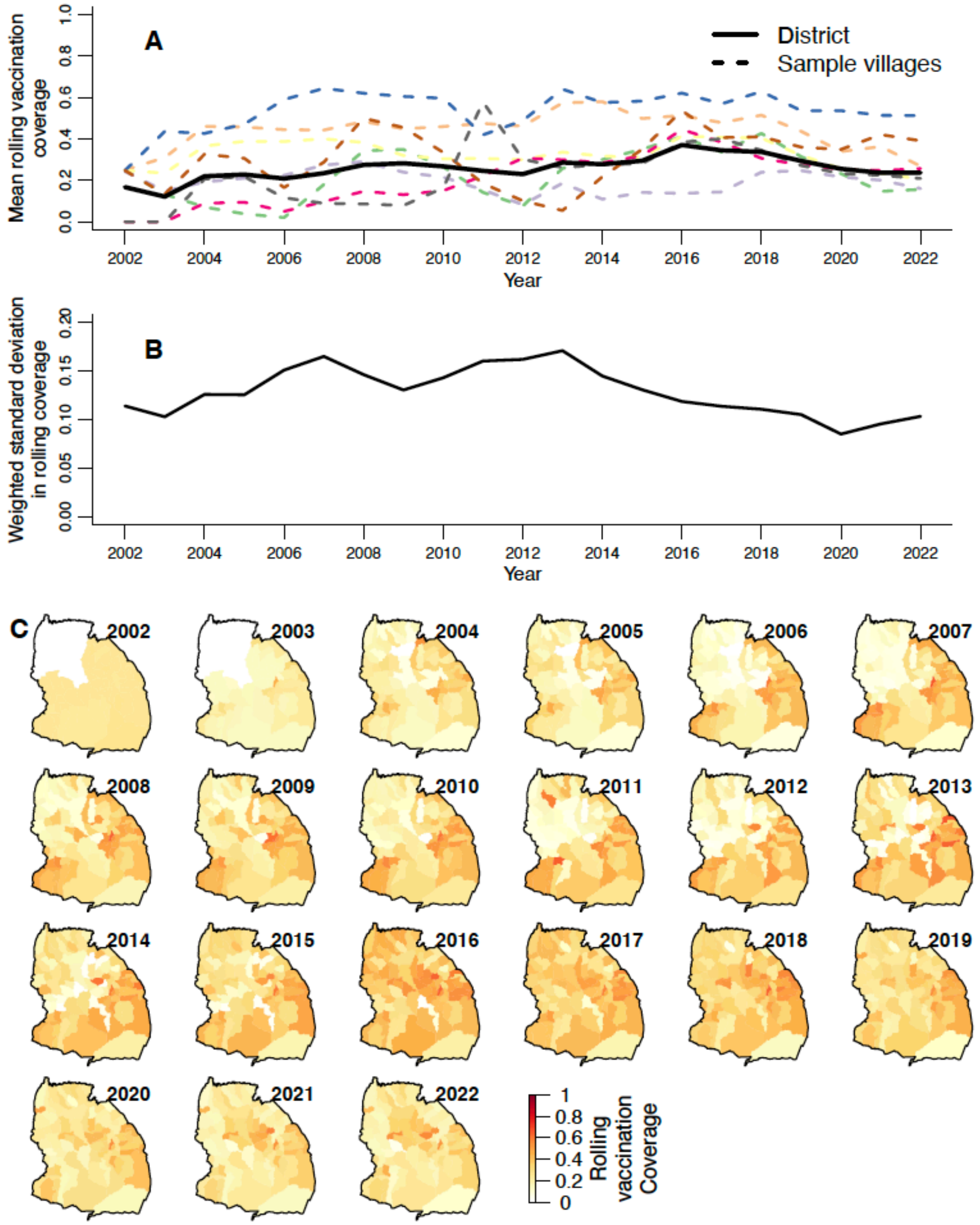

Figure S2. Mean of and spatial heterogeneity in rolling vaccination coverage each year. A) The mean rolling vaccination coverage in Serengeti District over each year (12-month averages of the values in Fig. 2C) is indicated by the solid black line. Mean rolling coverages each year for 8 randomly selected villages are indicated by dashed coloured lines. B) The weighted standard deviation in the rolling vaccination coverage over the villages in Serengeti District (yearly means of the values in Fig. 2C) . C) Mean rolling vaccination coverage in each village over each year from 2002-2022 is indicated by the colour scale.

bioRxiv preprint doi: https://doi.org/10.1101/2024.10.03.616420; this version posted January 16, 2025. The copyright holder for this preprint (which was not certified by peer review) is the author/funder, who has granted bioRxiv a license to display the preprint in perpetuity. It is made available under aCC-BY 4.0 International license.

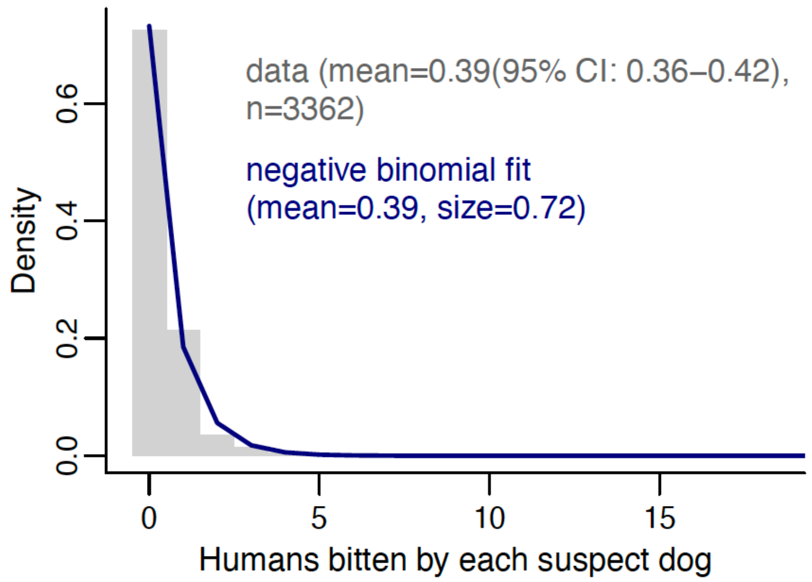

Figure S3: Human exposures per rabid dog. Histogram of human rabies exposures by each rabid dog from contact tracing data (grey bars), with fitted negative binomial distribution (blue line).

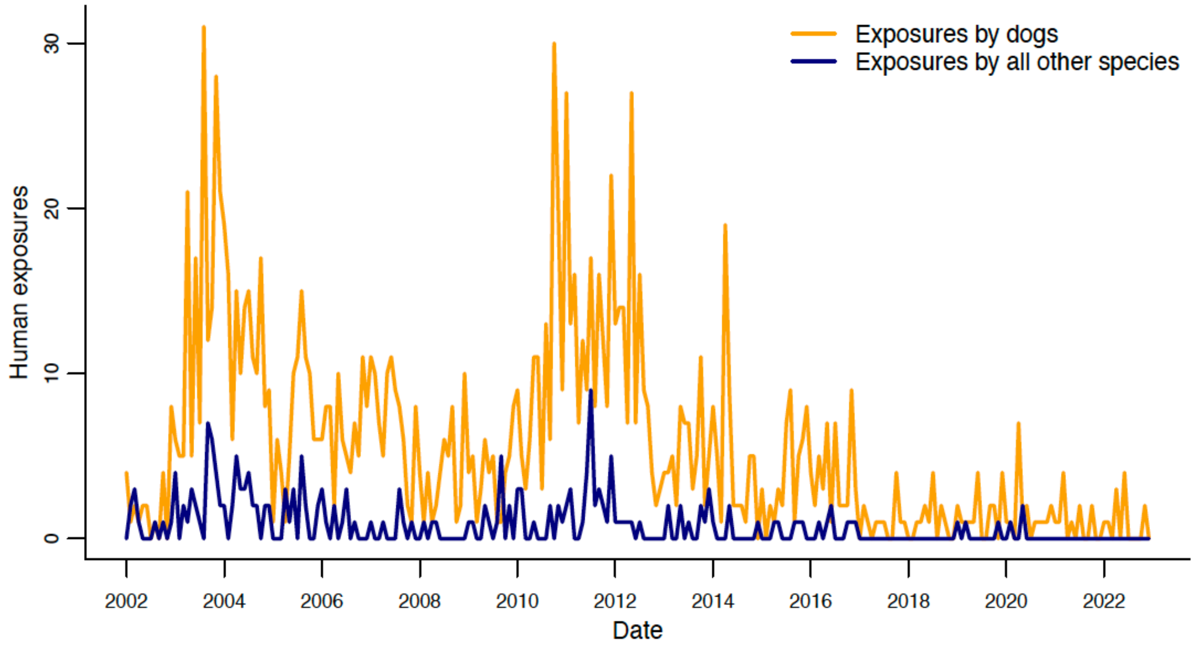

Figure S4: Probable human exposures by dogs vs. by all other species from contact tracing.

bioRxiv preprint doi: https://doi.org/10.1101/2024.10.03.616420; this version posted January 16, 2025. The copyright holder for this preprint (which was not certified by peer review) is the author/funder, who has granted bioRxiv a license to display the preprint in perpetuity. It is made available under aCC-BY 4.0 International license.

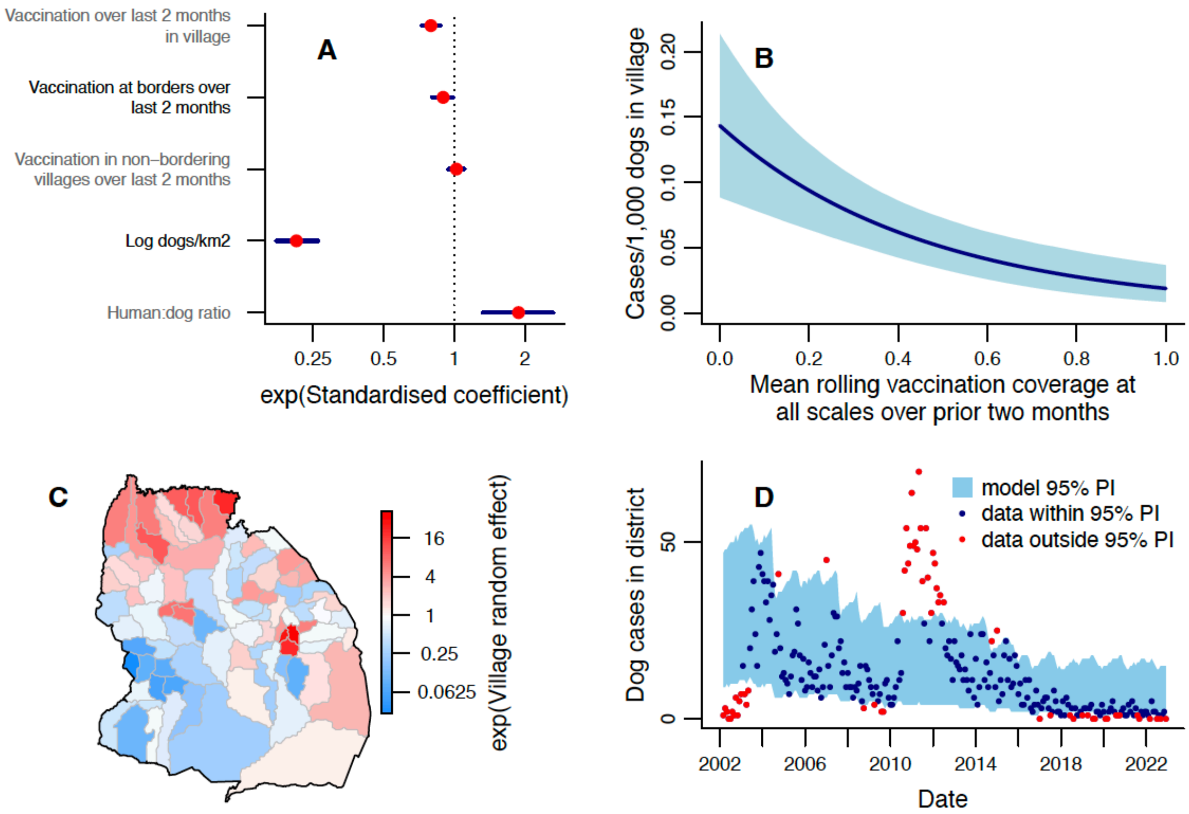

Figure S5: Impact of removing prior cases/dog explanatory variables from the monthly village-level GLMM for current cases per dog (compare with Fig. 3). A) Exponentiated standardised values of the coefficients estimated for each explanatory variable, with 95% CrIs. B) Line shows the expected cases/1,000 dogs (number of dog cases normalised by dog population) in a village this month for different mean rolling vaccination coverages across the focal village and district in the prior 2 months. Shaded areas show 95% credible intervals (CrIs), and predictions were obtained using average values of unspecified explanatory variables. C) Exponentiated random effect values for each village in the district. D) Comparison of observed monthly dog cases (points) with the 95% prediction interval from the fitted model. Data points in red fall outside the 95% prediction interval (PI).

bioRxiv preprint doi: https://doi.org/10.1101/2024.10.03.616420; this version posted January 16, 2025. The copyright holder for this preprint (which was not certified by peer review) is the author/funder, who has granted bioRxiv a license to display the preprint in perpetuity. It is made available under aCC-BY 4.0 International license.

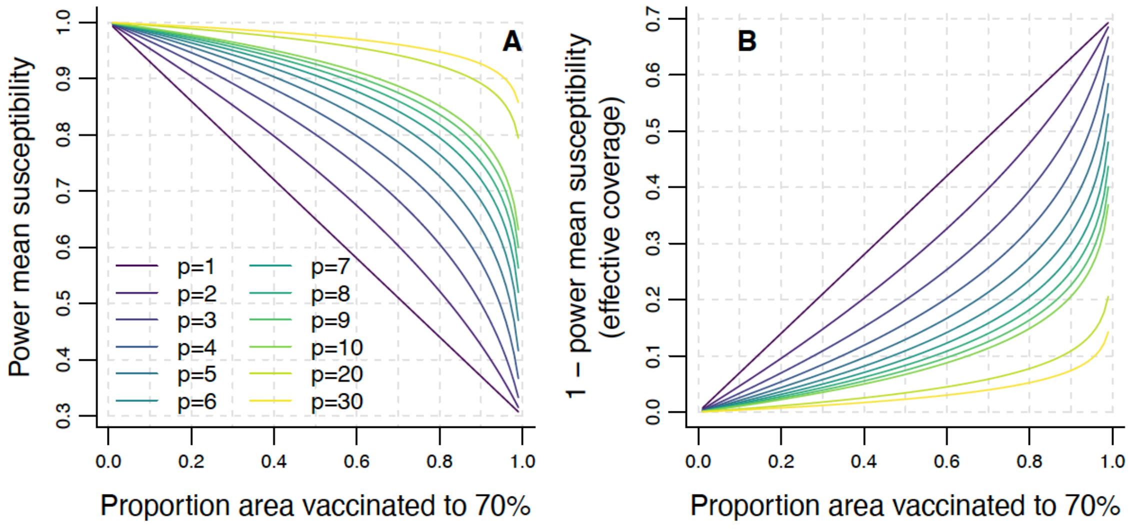

Figure S6: The impact of different powers on the power mean of susceptibility under different levels of heterogeneous vaccination. Here we assume a landscape where an increasing proportion of the area is vaccinated to 70% (30% susceptibility) while the remaining proportion remains at 0% coverage (100% susceptibility). We then calculate A) the power mean susceptibility and B) the effective coverage (1 – power mean susceptibility) at each proportion of area vaccinated at a range of values of the power p (equation (13)). p=1 is the arithmetic mean, and represents a scenario where, for a given proportion of dogs being vaccinated, the effective level of vaccination is the same regardless of how these vaccinated dogs are distributed over the area, i.e. heterogeneity in vaccination does not reduce (or increase) the impact of that vaccination on rabies cases. We used these curves showing the impact of different powers to select the prior distribution for $p \sim N(\mu=1, \sigma=2)$. If 99% of the area is covered, then the arithmetic mean coverage is 69.3%. If p=2, then the effective coverage for the heterogeneous landscape is 68.5%; 0.8% lower than if vaccination had been homogeneous. If p=5, however, effective coverage would be 58.4%, which is 10.9% below the arithmetic mean, despite only 1% of the area being uncovered, which seems an excessively large effect. The choice of σ=2 was therefore made to exclude $p \geq 5$ from the a priori confidence interval.

bioRxiv preprint doi: https://doi.org/10.1101/2024.10.03.616420; this version posted January 16, 2025. The copyright holder for this preprint (which was not certified by peer review) is the author/funder, who has granted bioRxiv a license to display the preprint in perpetuity. It is made available under aCC-BY 4.0 International license.

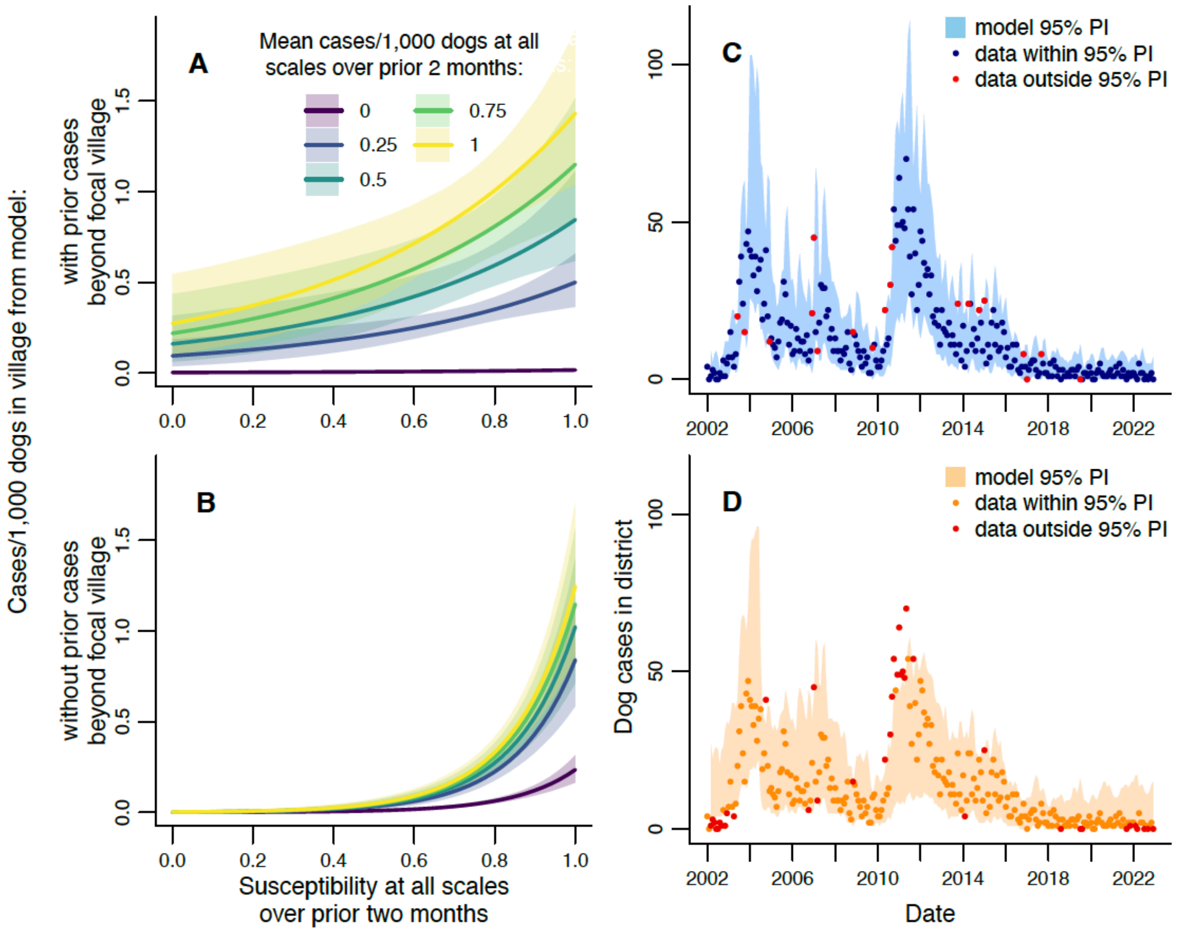

Figure S7: Impact of power mean susceptibility on rabies incidence at the village level and quality of model fits to data. A-B) Expected cases/1,000 dogs in a village from models with (A) and without (B) effects of prior incidence beyond the village. Predictions are shown for different mean susceptibilities (assuming homogeneous vaccination, i.e. power mean susceptibilities beyond the village equal susceptibility in the village) and mean cases/dog in the prior 2 months. Prior cases/dog values represent the observed district-level range and shaded areas show 95% CrIs, Predictions were obtained using average values of unspecified explanatory variables. C-D) Comparison of observed monthly dog cases (points) with the 95% prediction interval from the fitted model with (C) or without (D) prior incidence beyond the village. Data points in red fall outside the 95% prediction interval (PI).

bioRxiv preprint doi: https://doi.org/10.1101/2024.10.03.616420; this version posted January 16, 2025. The copyright holder for this preprint (which was not certified by peer review) is the author/funder, who has granted bioRxiv a license to display the preprint in perpetuity. It is made available under aCC-BY 4.0 International license.

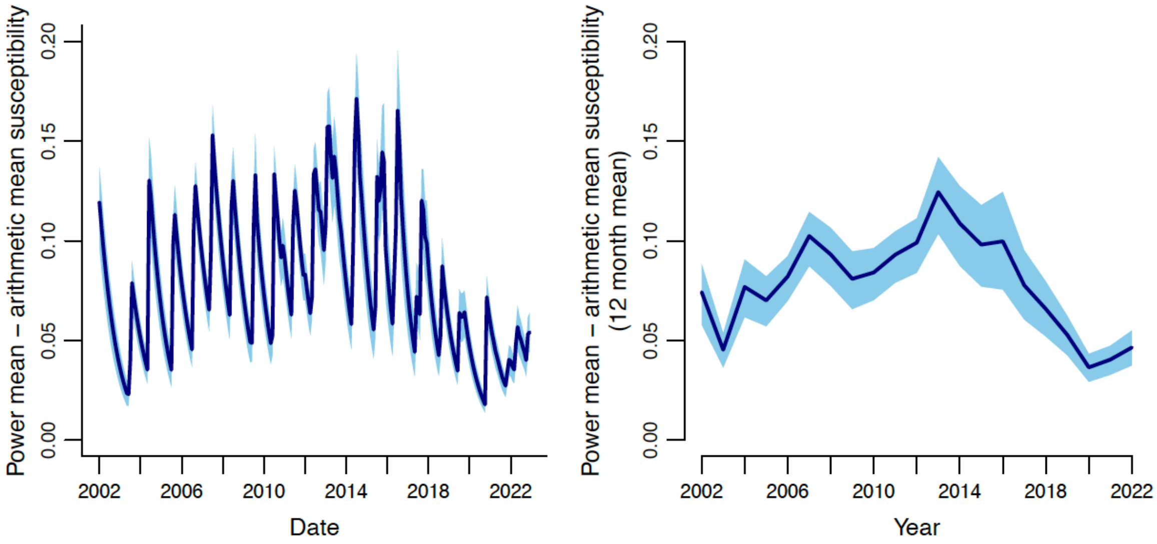

Figure S8: Difference between power mean and arithmetic mean susceptibility. Difference between power mean susceptibility calculated over all villages in the district using fitted values of p from the model without prior incidence beyond the focal village (Fig. 4C) minus the arithmetic mean for each month (A) or averaged over each year (B).

bioRxiv preprint doi: https://doi.org/10.1101/2024.10.03.616420; this version posted January 16, 2025. The copyright holder for this preprint (which was not certified by peer review) is the author/funder, who has granted bioRxiv a license to display the preprint in perpetuity. It is made available under aCC-BY 4.0 International license.

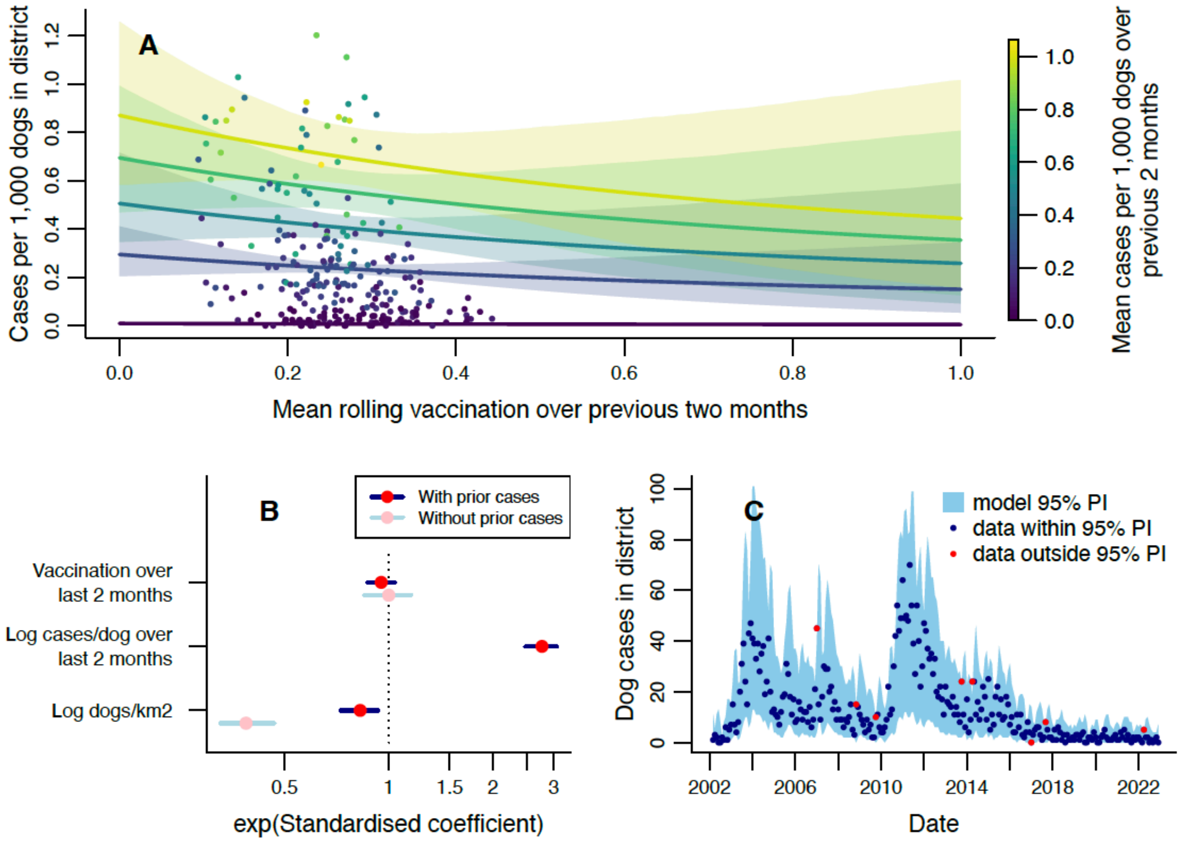

Figure S9: Modelling monthly dog rabies cases in Serengeti District at district level. A) Expected cases/dog (number of dog cases normalised by dog population) in the district this month for different mean rolling vaccination coverages and mean cases/dog in the prior 2 months. Shaded areas show 95% CrIs, points show the data, and predictions were obtained using the average value of dog density. B) Exponentiated standardised values of the coefficients estimated for each explanatory variable, with 95% CrIs. Coefficients obtained for a version of the model fitted without prior cases/dog as an explanatory variable are included for comparison. See Table S2 for tabulated parameter values. C) Comparison of observed monthly dog cases (points) with the 95% prediction interval from the fitted model. Data points in red fall outside the 95% prediction interval (PI).

bioRxiv preprint doi: https://doi.org/10.1101/2024.10.03.616420; this version posted January 16, 2025. The copyright holder for this preprint (which was not certified by peer review) is the author/funder, who has granted bioRxiv a license to display the preprint in perpetuity. It is made available under aCC-BY 4.0 International license.

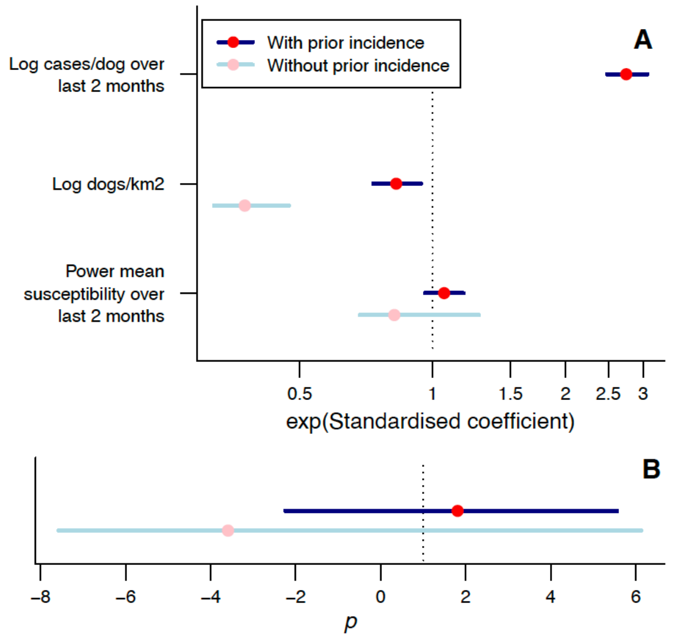

Figure S10: Using power mean susceptibility to model impacts of heterogeneity in rolling vaccination on district-level incidence. A) Exponentiated standardised estimated coefficients for each explanatory variable, with 95% CrIs. B) Estimated power p used to calculate power mean susceptibility. In A-B, estimates from models with and without effects of prior incidence are shown. See Table S2 for tabulated parameter values.

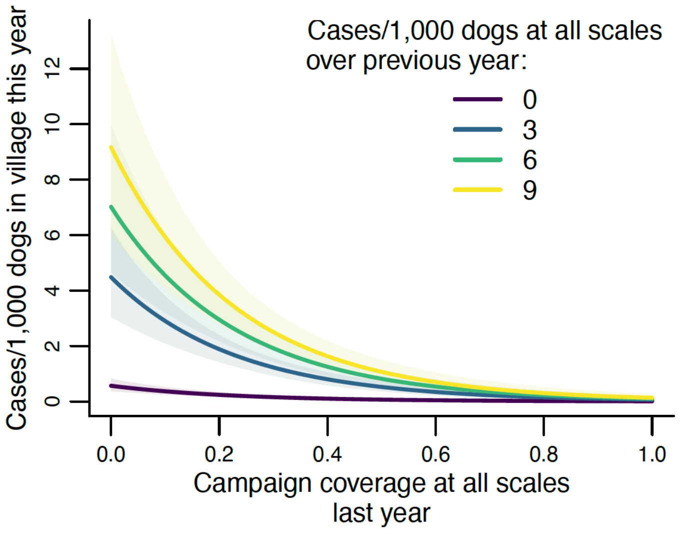

Figure S11: Annual village-level GLMM for current cases per dog. Lines show the expected cases per 1,000 dogs (number of dog cases normalised by dog population) in a village this year for different campaign vaccination coverages and cases per 1,000 dogs in the previous year. Prior incidence values were chosen to represent the range observed at district level. Shaded areas show 95% CrIs, and predictions were obtained using average values of unspecified explanatory variables. See Table S3 for tabulated parameter values.

bioRxiv preprint doi: https://doi.org/10.1101/2024.10.03.616420; this version posted January 16, 2025. The copyright holder for this preprint (which was not certified by peer review) is the author/funder, who has granted bioRxiv a license to display the preprint in perpetuity. It is made available under aCC-BY 4.0 International license.

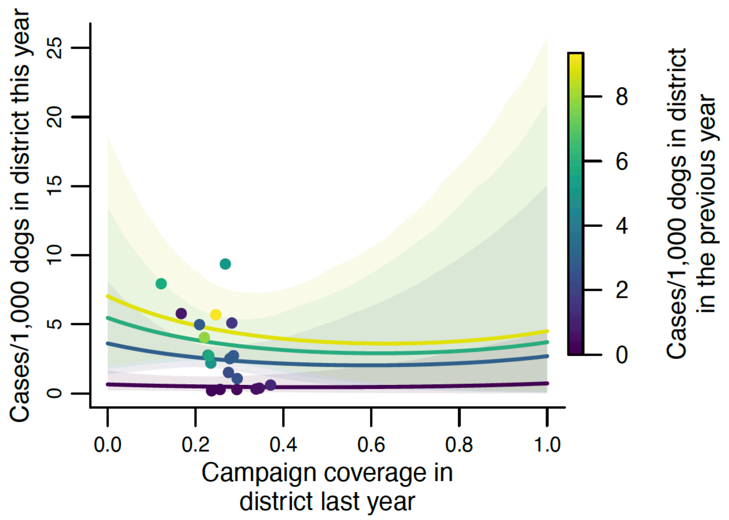

Figure S12: Annual district-level GLM for cases per dog. Lines show the expected cases per 1,000 dogs (number of dog cases normalised by dog population) in the district this year for different campaign vaccination coverages and cases per 1,000 dogs in the previous year. Prior incidence values were chosen to represent the range observed at district level. Shaded areas show 95% CrIs, and predictions were obtained using average values of unspecified explanatory variables. See Table S4 for tabulated parameter values.

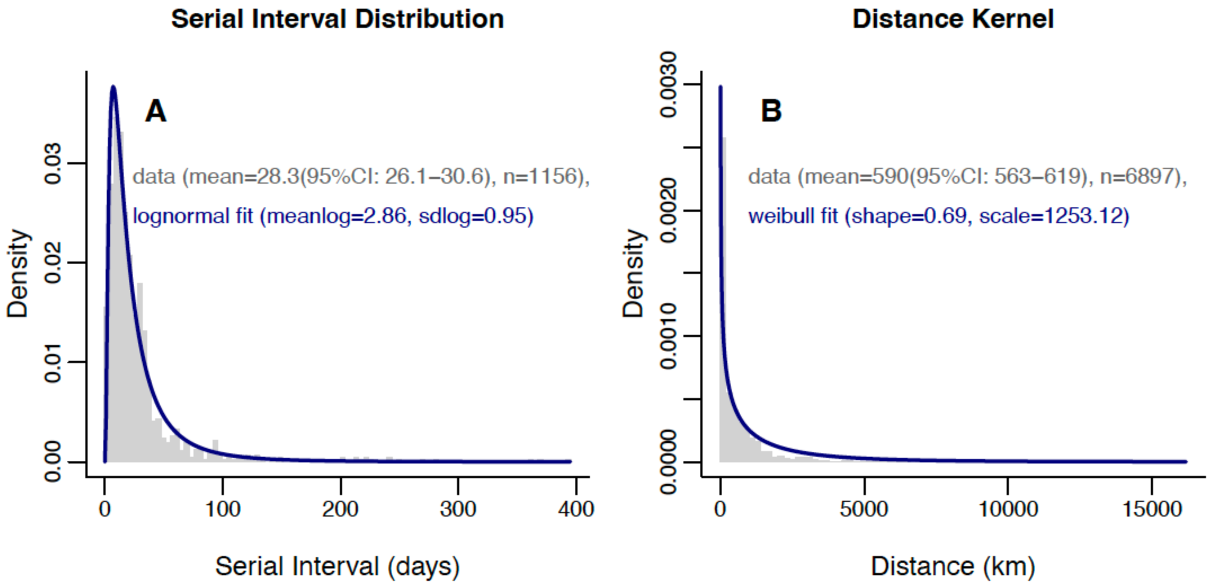

Figure S13: Serial interval distribution and distance kernel. A) Histogram of serial intervals calculated from contact tracing data (grey bars), with fitted lognormal distribution (blue line). B) Histogram of distances between the starting location of a case and the locations of its contacts (grey bars), with fitted weibull distribution (blue line).

bioRxiv preprint doi: https://doi.org/10.1101/2024.10.03.616420; this version posted January 16, 2025. The copyright holder for this preprint (which was not certified by peer review) is the author/funder, who has granted bioRxiv a license to display the preprint in perpetuity. It is made available under aCC-BY 4.0 International license.

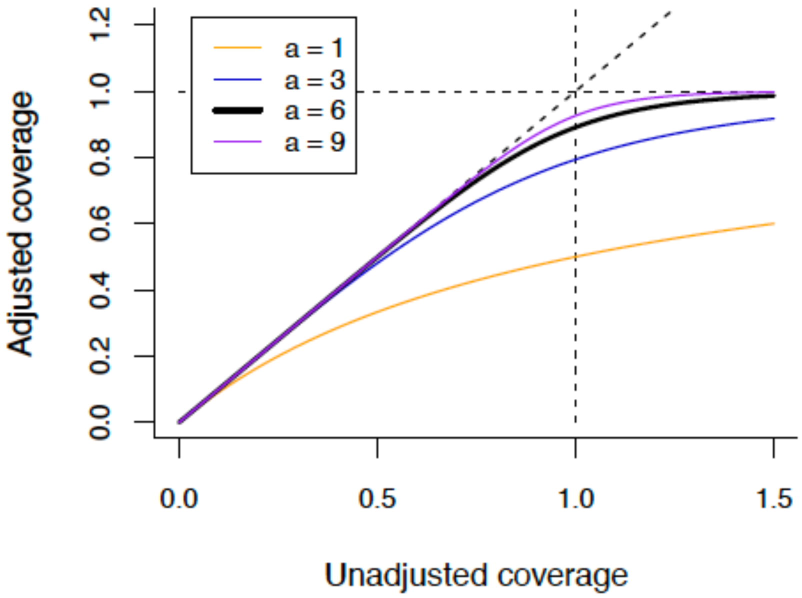

Figure S14: Bounding the proportion of dogs vaccinated between zero and one. Illustration of the function $b(x)=x/((1+x^a)^{1/a})$ used to bound coverage estimates below one. Throughout our analyses, we set a=6 when applying this function, but the impact of using alternative values is shown here.

Supplementary Tables

Table S1: Vaccination of Serengeti District by year. Numbers of dogs that received a vaccination in each year, campaign coverage (percentage of the district dog population vaccinated in campaigns each year), and campaign completeness (percentage of villages in the district that held a campaign in each year).

	**2002**	**2003**	**2004**	**2005**	**2006**	**2007**	**2008**	**2009**	**2010**	**2011**	**2012**	**2013**	**2014**	**2015**	**2016**	**2017**	**2018**	**2019**	**2020**	**2021**	**2022**
Number of dogs that received a vaccination	0	4,199	13,126	7,675	7,433	11,562	14,016	14,098	11,622	8,252	12,532	16,229	19,288	21,150	22,724	26,419	11,050	19,305	14,882	6,343	17,036
Campaign coverage	0	9	28	16	15	23	26	26	20	14	21	26	30	32	33	37	15	25	19	8	20
Campaign completeness	0	48	90	56	50	86	97	97	62	50	65	72	86	97	95	97	50	100	99	44	92

Table S2: Parameter estimates for monthly district-level GLMs for cases/dog in the district in the current month. 95% credible intervals (CrIs) in brackets. Coefficients for fixed effects where the 95% CrI does not include zero are marked *. Predictions from model 1 (and coefficients for model 2) are presented in Fig. S9). Parameters from models 3 and 4 are illustrated in Fig. S10. Vaccination, susceptibility and cases/dog variables are all averages over the prior two months.

**Parameter**	**1. Full model without power mean**	**2. Without power mean or prior cases/dog**	**3. Full model with power mean**	**4. Power mean without prior cases/dog**
Intercept	1.52 (0, 3.1)	6.67 (4.03, 9.31)	0.5 (-2.15, 2.99)	8.01 (0.17, 11.42)
Rolling vaccination coverage	-0.75 (-2.06, 0.55)	-0.06 (-2.18, 2.13)		
Susceptibility			0.92 (-0.5, 2.46)	-1.32 (-2.98, 4.19)
Log cases/dog	0.78 (0.7, 0.86)*		0.78 (0.7, 0.86)*	
Log dogs/km^2	-0.98 (-1.59, -0.38)*	-4.71 (-5.59, -3.81)*	-0.94 (-1.54, -0.35)*	-4.89 (-5.72, -3.68)*
size (negative binomial distribution parameter)	4.76 (3.55, 6.32)	1.3 (1.06, 1.57)	4.79 (3.58, 6.31)	1.36 (1.11, 1.65)
p (power used in calculating power means)			2.02 (-1.74, 5.73)	-3.06 (-7.32, 6.32)

bioRxiv preprint doi: https://doi.org/10.1101/2024.10.03.616420; this version posted January 16, 2025. The copyright holder for this preprint (which was not certified by peer review) is the author/funder, who has granted bioRxiv a license to display the preprint in perpetuity. It is made available under aCC-BY 4.0 International license.

Table S3: Coefficients for the annual village-level negative binomial GLMMs. 95% credible intervals in brackets. Coefficients for fixed effects where the 95% CrI does not include zero are marked *. Predictions from the model 4 (including campaign coverage and cases/dog in the last year as explanatory variables) are presented in Fig. S11.

Coefficient	1. Model with campaign coverage last year	2. Model with mean campaign coverage over the last 2 years	3. Model with mean campaign coverage over the last 3 years	4. Model with campaign coverage and incidence last year
Intercept	1.37 (-0.96, 3.98)	-0.02 (-2.35, 2.73)	-0.62 (-2.97 - 2.17)	1.5 (-0.03, 3.13)
Campaign coverage last year in the focal village	-1.12 (-1.8, -0.43)*			-1.07 (-1.73, -0.38)*
Campaign coverage last year in bordering villages	-0.44 (-1.54, 0.65)			-0.5 (-1.56, 0.56)
Campaign coverage last year in non-bordering villages	-0.81 (-2.35, 0.66)			-2.73 (-4.09, -1.37)*
Mean campaign coverage over the last 2 years in the focal village		-1.32 (-2.25, -0.34)*		
Mean campaign coverage over the last 2 years in bordering villages		-1.38 (-2.92, 0.15)		
Mean campaign coverage over the last 2 years in non-bordering villages		-1 (-3.08, 1.08)		
Mean campaign coverage over the last 3 years in the focal village			-1.48 (-2.63, -0.29)*	
Mean campaign coverage over the last 3 years in bordering villages			-1.65 (-3.51, 0.2)	
Mean campaign coverage over the last 3 years in non-bordering villages			-0.42 (-3.28, 2.45)	
Log cases/dog last year in the focal village				0.08 (0.02, 0.14)*
Log cases/dog last year in bordering villages				0.16 (0.07, 0.26)*
Log cases/dog last year in non-bordering villages				0.43 (0.29, 0.56)*
Log dogs/km^2	-2.9 (-3.67, -2.2)*	-2.44 (-3.29, -1.68)*	-2.28 (-3.22, -1.52)*	-1.37 (-1.9, -0.92)*
Human:dog ratio	0.53 (0.22, 0.85)*	0.56 (0.28, 0.87)*	0.58 (0.28, 0.87)*	0.38 (0.18, 0.58)*
Standard deviation of village random effect	1.69 (1.28, 2.16)	1.5 (1.11, 1.97)	1.44 (1.05, 1.94)	1 (0.76, 1.3)
size (negative binomial distribution parameter)	0.43 (0.38, 0.49)	0.41 (0.36, 0.47)	0.38 (0.33, 0.44)	0.49 (0.42, 0.55)

Table S4: Coefficients for the annual district-level negative binomial GLMs. 95% credible intervals in brackets. Coefficients for fixed effects where the 95% CrI does not include zero are marked *. Predictions from model 4 (including campaign coverage and cases/dog in the last year as explanatory variables) are presented in Fig. S12.

**Coefficient**	**1. Model with campaign coverage last year**	**2. Model with mean campaign coverage over the last 2 years**	**3. Model with mean campaign coverage over the last 3 years**	**4. Model with campaign coverage and incidence last year**
Intercept	12.29 (4.84, 19.74)	13.8 (5.6, 22.2)	14.64 (5.61, 23.25)	9.51 (4, 15.09)
Campaign coverage last year	0.55 (-4.1, 4.93)			-1.08 (-4.66, 2.42)
Mean campaign coverage over the last 2 years		0.6 (-5.75, 6.87)		
Mean campaign coverage over the last 3 years			1.92 (-6.96, 10.67)	
Log cases/dog last year				0.55 (0.21, 0.89)*
Log dogs/km^2	-5.7 (-8.04, -3.29)*	-6.15 (-8.79, -3.45)*	-6.5 (-9.16, -3.6)*	-3.72 (-5.84, -1.64)*
size (negative binomial distribution parameter)	2.08 (1.03, 3.6)	2.08 (0.99, 3.59)	2.03 (0.94, 3.56)	3.25 (1.5, 5.81)

Table S5: Fitted distributions for incubation period (units=days), serial interval (units=days) and distance kernel (units=metres). For each epidemiological variable calculated from the contact tracing data with sample size n, we fitted gamma, lognormal and Weibull distributions. Estimates of the parameters for each distribution are provided. The best fitting model for each epidemiological variable based on AIC is highlighted in bold. The 95th, 97.5th and 99th percentiles of each distribution are given.

	n	Distribution	Parameter 1: Name	Parameter 1: Value (95%CI)	Parameter 2: Name	Parameter 2: Value (95%CI)	AIC	95th percentile	97.5th percentile	99th percentile
**Incubation Period**	1,212	Gamma	shape	1.18 (1.1, 1.26)	rate	0.04 (0.04, 0.05)	10421	77.2	93.9	115.9
		**Lognormal**	**meanlog**	**2.82 (2.77, 2.87)**	**sdlog**	**0.95 (0.91, 0.99)**	**10159**	**80.1**	**108.0**	**152.9**
		Weibull	shape	1 (0.96, 1.05)	scale	27.27 (25.78, 28.89)	10440	81.6	100.5	125.4
**Serial Interval**	1,156	Gamma	shape	1.18 (1.1, 1.27)	rate	0.04 (0.04, 0.05)	10022	79.9	97.2	119.9
		**Lognormal**	**meanlog**	**2.86 (2.81, 2.92)**	**sdlog**	**0.95 (0.91, 0.99)**	**9781**	**83.4**	**112.5**	**159.3**
		Weibull	shape	1 (0.96, 1.05)	scale	28.29 (26.73, 30.09)	10041	84.5	104.0	129.8
**Distance kernel**	6,897	Gamma	shape	0.58 (0.55, 0.6)	rate	0.00038 (0.00035, 0.00041)	45177	5596.7	7216.4	9407.9
		Lognormal	meanlog	6.45 (6.4, 6.49)	sdlog	1.74 (1.69, 1.8)	45321	11129.8	19284.6	36540.9
		**Weibull**	**shape**	**0.69 (0.67, 0.71)**	**scale**	**1253.12 (1192.2, 1315.93)**	**45143**	**6142.8**	**8304.8**	**11453.2**
