# Supplementary material for: Reducing spatial heterogeneity in coverage improves the effectiveness of dog vaccination against rabies
Source: bioRxiv. 2024 Oct 12:2024.10.03.616420. Preprint. [Version 2] doi: 10.1101/2024.10.03.616420 (PMC11482771; doi:10.1101/2024.10.03.616420)
Supplement: Supplementary file 1 [file NIHPP2024.10.03.616420v2-supplement-1.pdf]

bioRxiv preprint doi: https://doi.org/10.1101/2024.10.03.616420; this version posted October 13, 2024. The copyright holder for this preprint (which was not certified by peer review) is the author/funder, who has granted bioRxiv a license to display the preprint in perpetuity. It is made available under aCC-BY 4.0 International license.

Supplementary information

Supplementary Videos

Video S1

Video S1: Monthly spatial distribution of dog cases, human exposures and dog vaccination coverage over the study period. Estimated vaccination coverage at the village-level for each month in 2002-2022 is indicated by the colour scale. Locations of dog cases (blue points), human exposures (purple triangles) and human deaths (large black triangles) each month are indicated.

bioRxiv preprint doi: https://doi.org/10.1101/2024.10.03.616420; this version posted October 13, 2024. The copyright holder for this preprint (which was not certified by peer review) is the author/funder, who has granted bioRxiv a license to display the preprint in perpetuity. It is made available under aCC-BY 4.0 International license.

Supplementary Figures

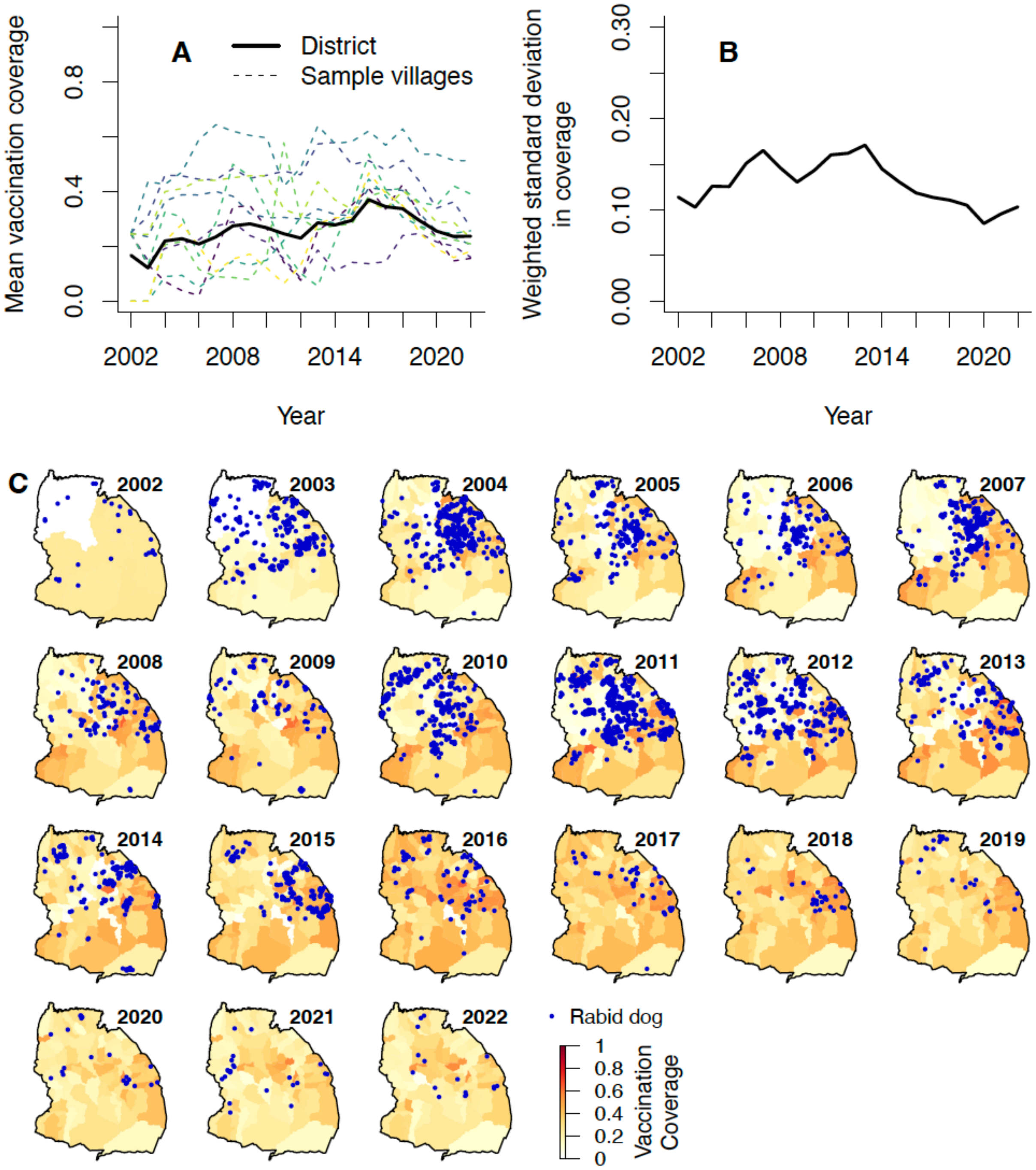

Figure S1. Mean vaccination coverage each year. A) The mean vaccination coverage in Serengeti District over each year (12-month averages of the values in Fig. 2B) is indicated by the solid black line. Mean vaccination coverages each year for 10 randomly selected villages are indicated by dashed coloured lines. B) The weighted standard deviation in vaccination coverage over the villages in Serengeti District (yearly means of the values in Fig. 2B) . C) Mean estimated vaccination coverage in each village over each year from 2002-2022 is indicated by the colour scale. Locations of dog cases in each year are indicated by blue points.

bioRxiv preprint doi: https://doi.org/10.1101/2024.10.03.616420; this version posted October 13, 2024. The copyright holder for this preprint (which was not certified by peer review) is the author/funder, who has granted bioRxiv a license to display the preprint in perpetuity. It is made available under aCC-BY 4.0 International license.

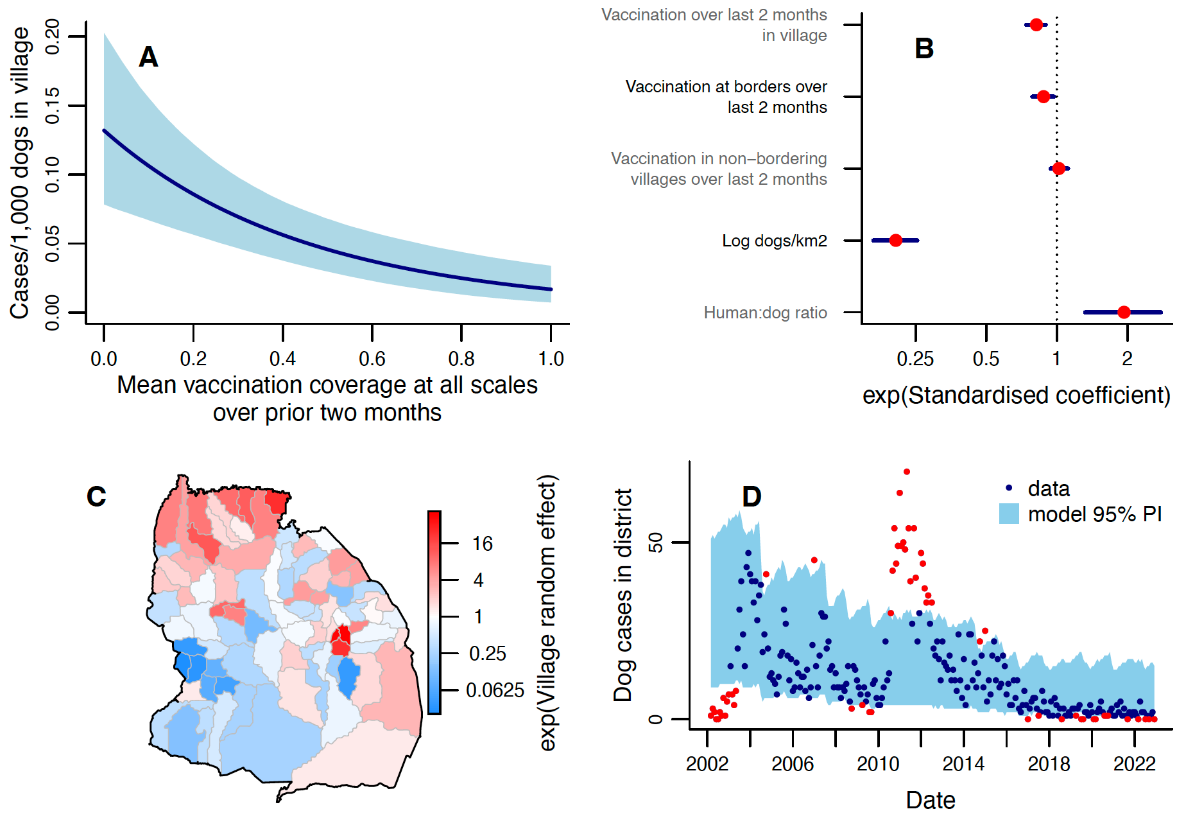

Figure S2: Impact of removing prior cases/dog explanatory variables from the monthly village-level GLMM for current cases per dog (compare with Fig. 3). A) Line shows the expected cases/1,000 dogs (number of dog cases normalised by dog population) in a village this month for different mean vaccination coverages across the focal village and district in the prior 2 months. Shaded areas show 95% credible intervals (CrIs), and predictions were obtained using average values of unspecified explanatory variables. B) Exponentiated standardised values of the coefficients estimated for each explanatory variable, with 95% CrIs. C) Exponentiated random effect values for each village in the district. D) Comparison of observed monthly dog cases (points) with the 95% prediction interval from the fitted model. Data points in red fall outside the 95% prediction interval (PI).

bioRxiv preprint doi: https://doi.org/10.1101/2024.10.03.616420; this version posted October 13, 2024. The copyright holder for this preprint (which was not certified by peer review) is the author/funder, who has granted bioRxiv a license to display the preprint in perpetuity. It is made available under aCC-BY 4.0 International license.

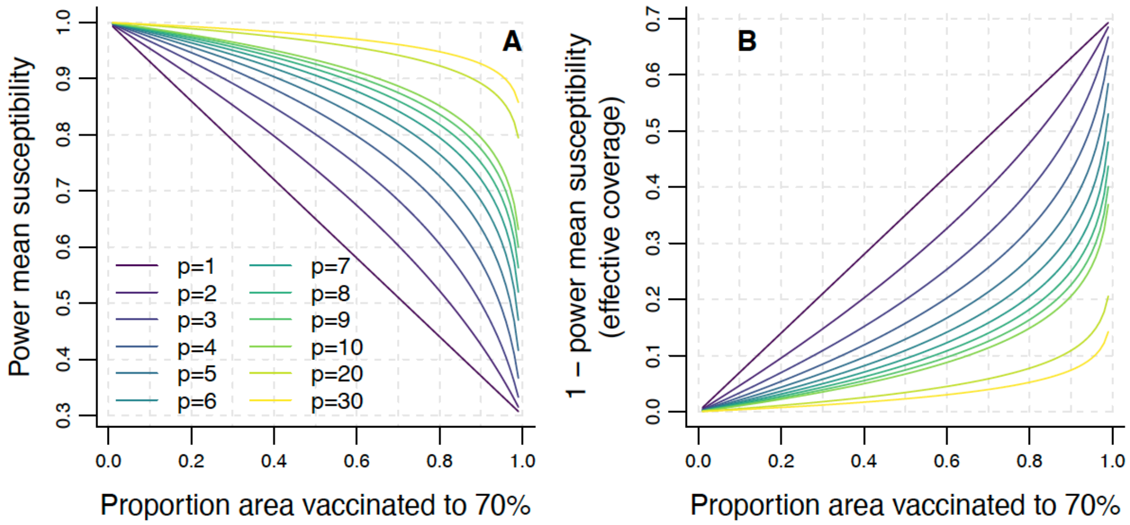

Figure S3: The impact of different powers on the power mean of susceptibility under different levels of heterogeneous vaccination. Here we assume a landscape where an increasing proportion of the area is vaccinated to 70% (30% susceptibility) while the remaining proportion remains at 0% coverage (100% susceptibility). We then calculate A) the power mean susceptibility and B) the effective coverage (1 – power mean susceptibility) at each proportion of area vaccinated at a range of values of the power p (equation (13)). p=1 is the arithmetic mean, and represents a scenario where, for a given proportion of dogs being vaccinated, the effective level of vaccination is the same regardless of how these vaccinated dogs are distributed over the area, i.e. heterogeneity in vaccination does not reduce (or increase) the impact of that vaccination on rabies cases. We used these curves showing the impact of different powers to select the prior distribution for $p \sim N(\mu=1, \sigma=2)$. If 99% of the area is covered, then the arithmetic mean coverage is 69.3%. If p=2, then the effective coverage for the heterogeneous landscape is 68.5%; 0.8% lower than if vaccination had been homogeneous. If p=5, however, effective coverage would be 58.4%, which is 10.9% below the arithmetic mean, despite only 1% of the area being uncovered, which seems an excessively large effect. The choice of σ=2 was therefore made to exclude $p \geq 5$ from the a priori confidence interval.

bioRxiv preprint doi: https://doi.org/10.1101/2024.10.03.616420; this version posted October 13, 2024. The copyright holder for this preprint (which was not certified by peer review) is the author/funder, who has granted bioRxiv a license to display the preprint in perpetuity. It is made available under aCC-BY 4.0 International license.

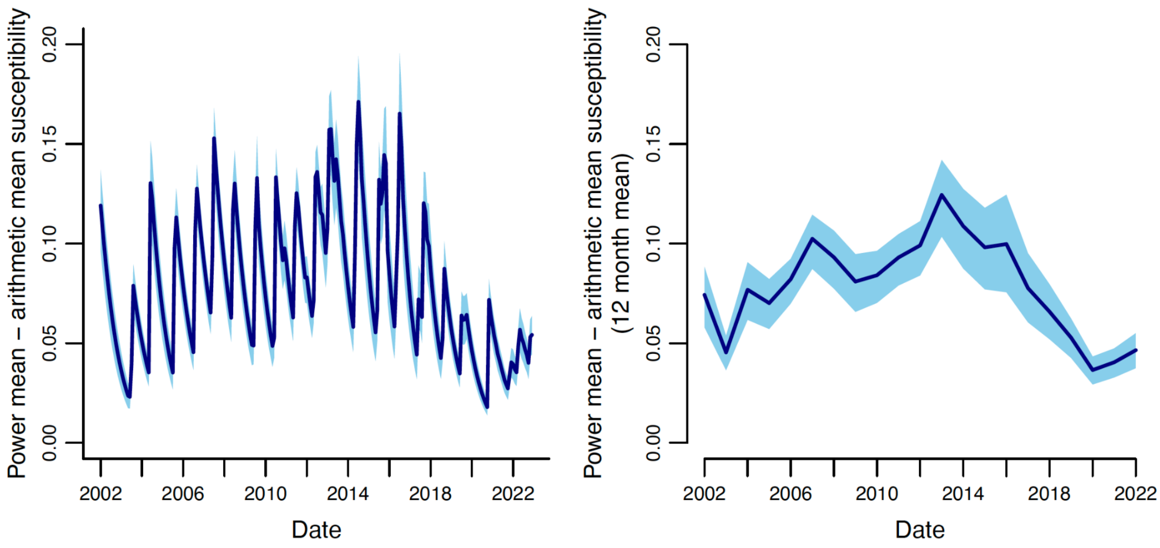

Figure S4: Difference between power mean and arithmetic mean susceptibility. Difference between power mean susceptibility calculated over all villages in the district using fitted values of *p* from the model without prior incidence beyond the focal village (Fig. 4C) minus the arithmetic mean for each month (A) or averaged over each year (B).

bioRxiv preprint doi: https://doi.org/10.1101/2024.10.03.616420; this version posted October 13, 2024. The copyright holder for this preprint (which was not certified by peer review) is the author/funder, who has granted bioRxiv a license to display the preprint in perpetuity. It is made available under aCC-BY 4.0 International license.

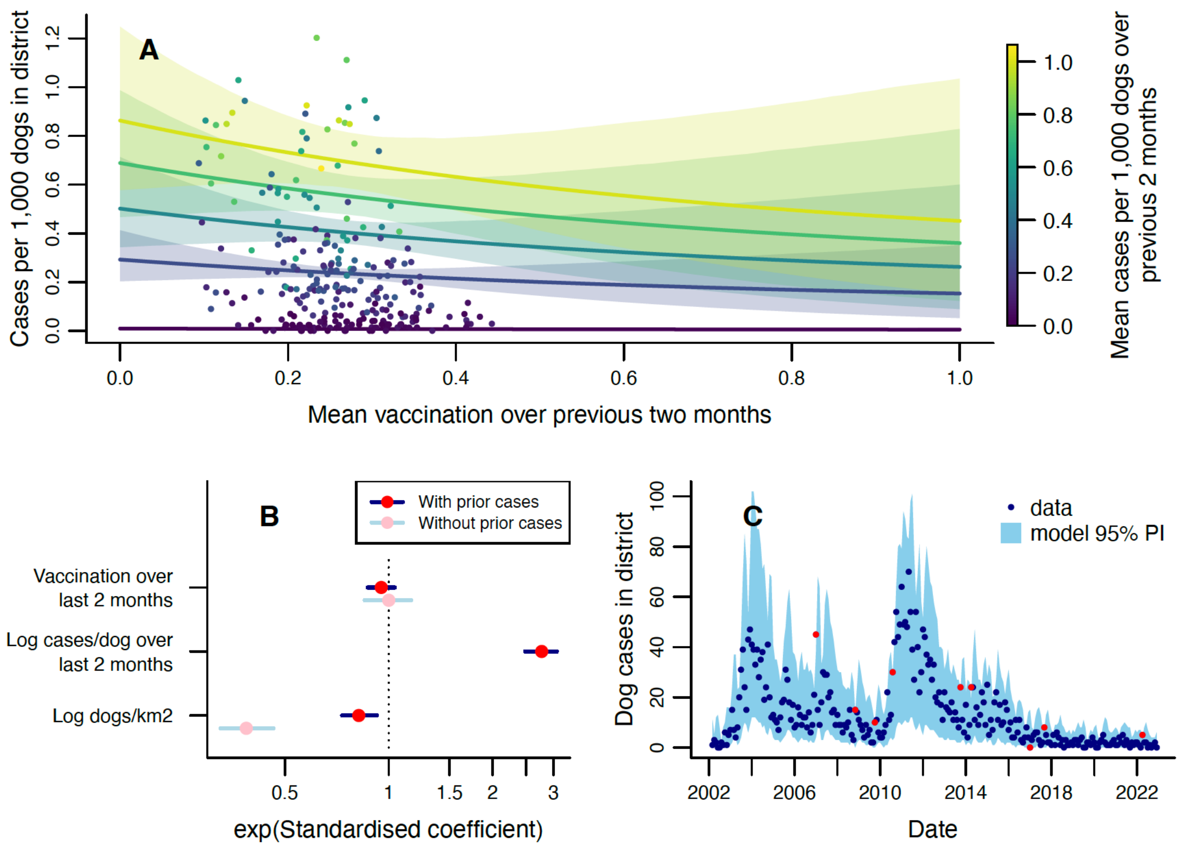

Figure S5: Modelling monthly dog rabies cases in Serengeti District at district level. A) Expected cases/dog (number of dog cases normalised by dog population) in the district this month for different mean vaccination coverages and mean cases/dog in the prior 2 months. Shaded areas show 95% CrIs, points show the data, and predictions were obtained using the average value of dog density. B) Exponentiated standardised values of the coefficients estimated for each explanatory variable, with 95% CrIs. Coefficients obtained for a version of the model fitted without prior cases/dog as an explanatory variable are included for comparison. See Table S3 for tabulated parameter values. C) Comparison of observed monthly dog cases (points) with the 95% prediction interval from the fitted model. Data points in red fall outside the 95% prediction interval (PI).

bioRxiv preprint doi: https://doi.org/10.1101/2024.10.03.616420; this version posted October 13, 2024. The copyright holder for this preprint (which was not certified by peer review) is the author/funder, who has granted bioRxiv a license to display the preprint in perpetuity. It is made available under aCC-BY 4.0 International license.

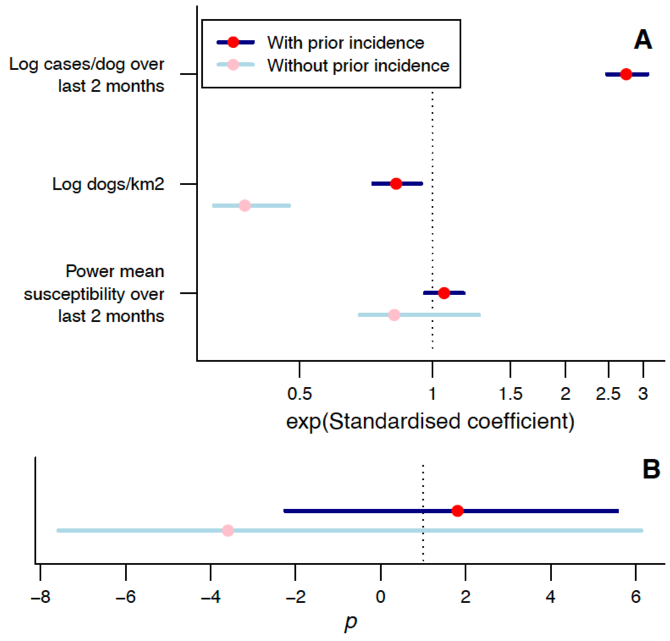

Figure S6: Using power mean susceptibility to model impacts of heterogeneity in vaccination on district-level incidence. A) Exponentiated standardised estimated coefficients for each explanatory variable, with 95% CrIs. B) Estimated power *p* used to calculate power mean susceptibility. In A-B, estimates from models with and without effects of prior incidence are shown. See Table S3 for tabulated parameter values.

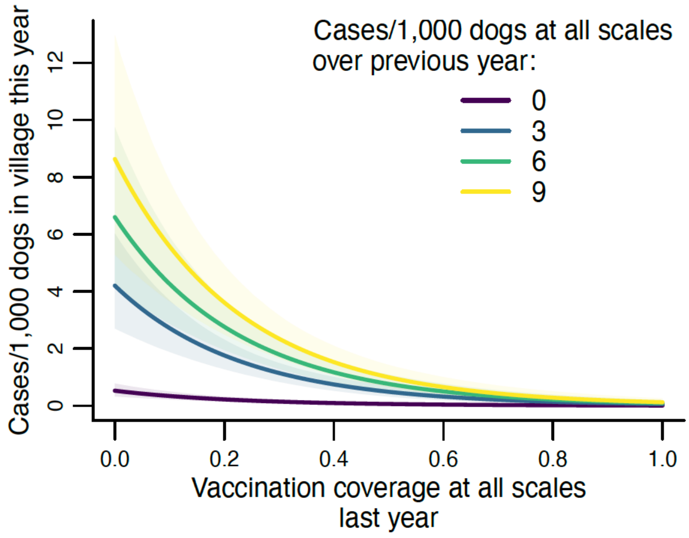

Figure S7: Annual village-level GLMM for current cases per dog. Lines show the expected cases per 1,000 dogs (number of dog cases normalised by dog population) in a village this year for different vaccination coverages and cases per 1,000 dogs in the previous year. Prior incidence values were chosen to represent the range observed at district level. Shaded areas show 95% CrIs, and predictions were obtained using average values of unspecified explanatory variables. See supplementary Table S4 for tabulated parameter values.

bioRxiv preprint doi: https://doi.org/10.1101/2024.10.03.616420; this version posted October 13, 2024. The copyright holder for this preprint (which was not certified by peer review) is the author/funder, who has granted bioRxiv a license to display the preprint in perpetuity. It is made available under aCC-BY 4.0 International license.

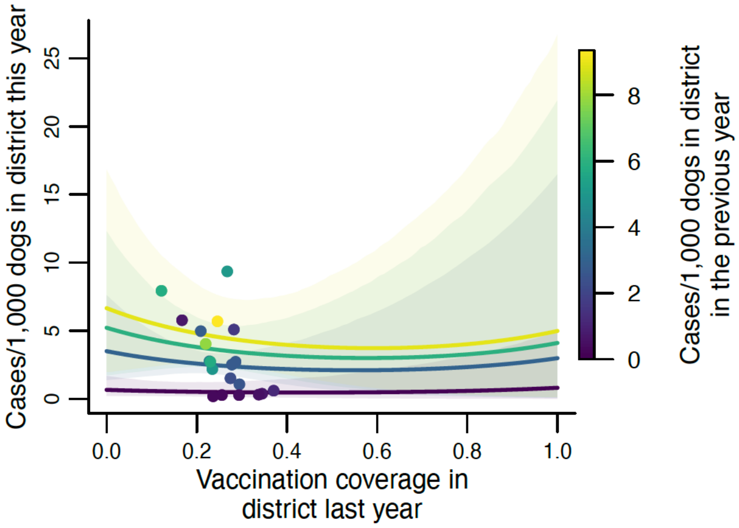

Figure S8: Annual district-level GLM for cases per dog. Lines show the expected cases per 1,000 dogs (number of dog cases normalised by dog population) in the district this year for different vaccination coverages and cases per 1,000 dogs in the previous year. Prior incidence values were chosen to represent the range observed at district level. Shaded areas show 95% CrIs, and predictions were obtained using average values of unspecified explanatory variables. See supplementary Table S5 for tabulated parameter values.

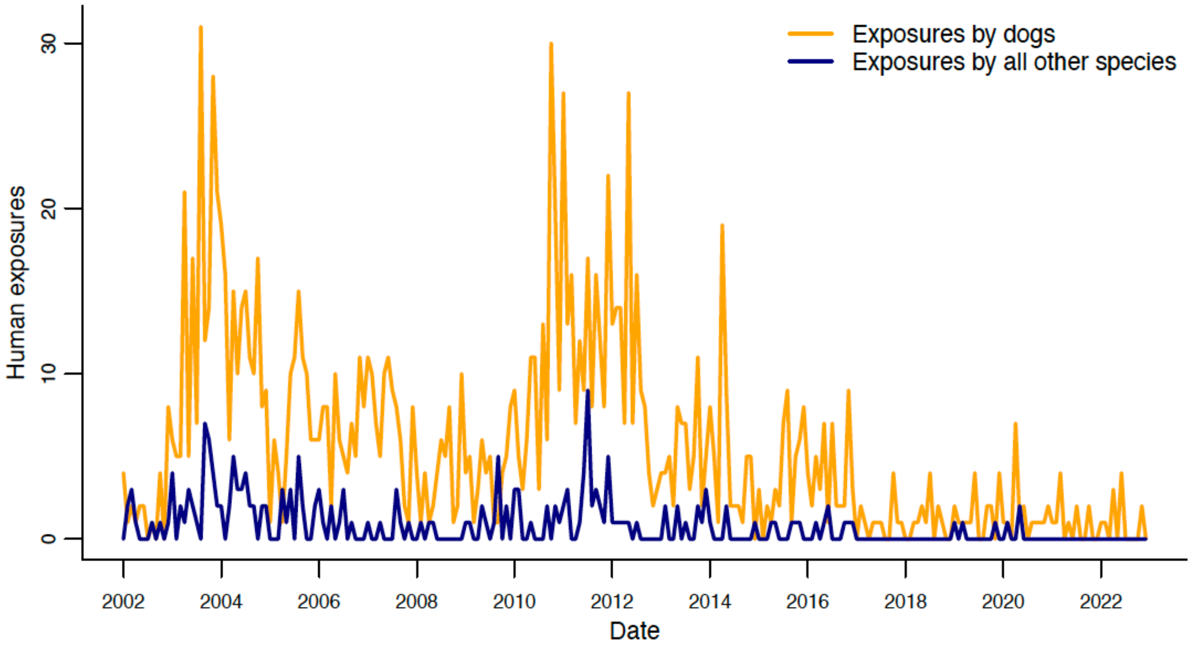

Figure S9: Probable human exposures by dogs vs. by all other species from contact tracing.

bioRxiv preprint doi: https://doi.org/10.1101/2024.10.03.616420; this version posted October 13, 2024. The copyright holder for this preprint (which was not certified by peer review) is the author/funder, who has granted bioRxiv a license to display the preprint in perpetuity. It is made available under aCC-BY 4.0 International license.

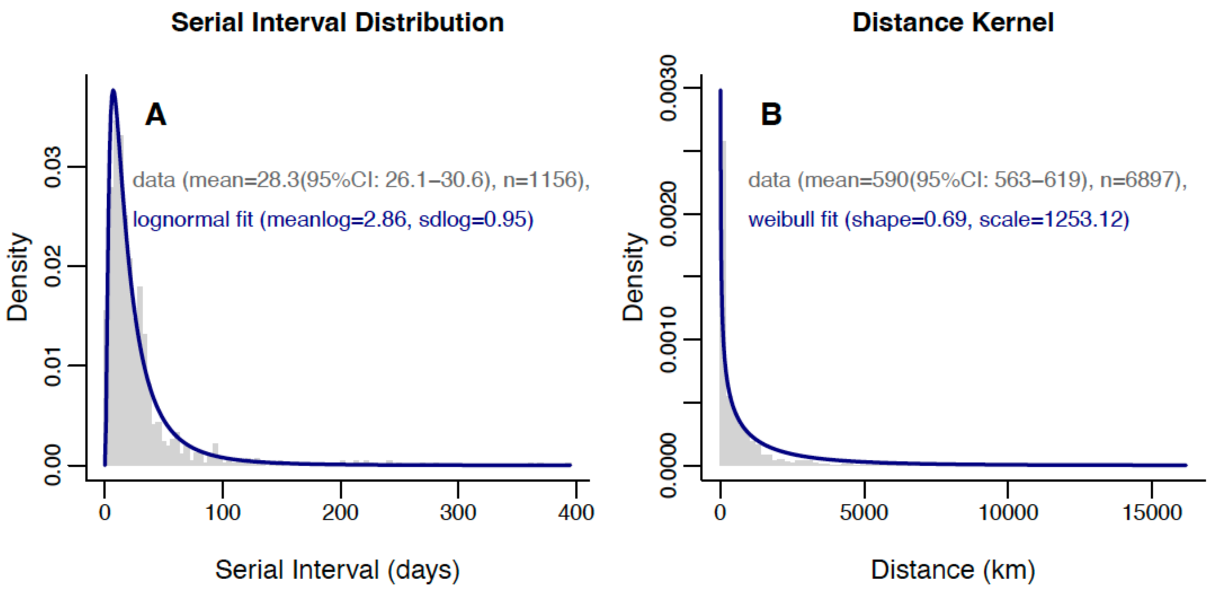

Figure S10: Serial interval distribution and distance kernel. A) Histogram of serial intervals calculated from contact tracing data (grey bars), with fitted lognormal distribution (blue line). B) Histogram of distances between the starting location of a case and the locations of its contacts (grey bars), with fitted weibull distribution (blue line).

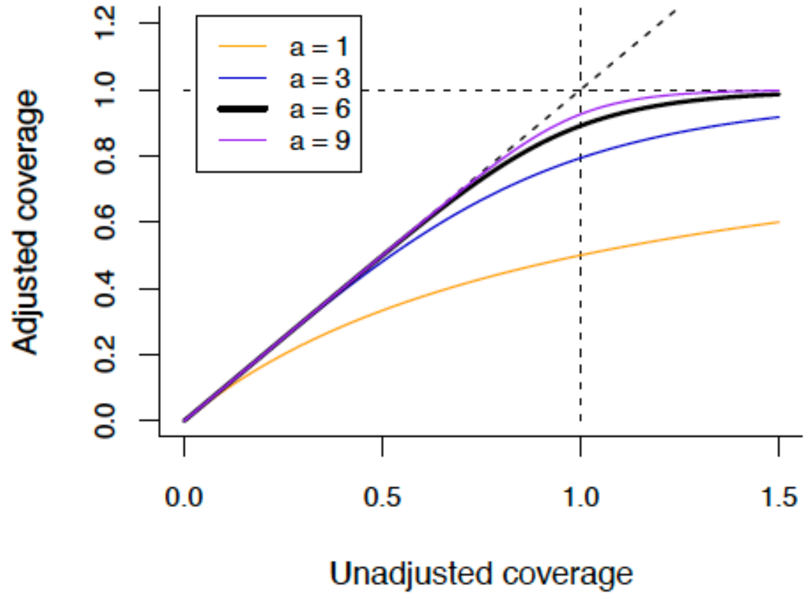

Figure S11: Bounding the proportion of dogs vaccinated between zero and one. Illustration of the function $b(x)=x/((1+x^a)^{1/a})$ used to bound coverage estimates below one. Throughout our analyses, we set a=6 when applying this function, but the impact of using alternative values is shown here.

Supplementary Tables

Table S1: Vaccination of Serengeti District by year. Numbers of dogs vaccinated, percentage of the district dog population vaccinated, and percentage of villages where a campaign was conducted in each year.

	2002	2003	2004	2005	2006	2007	2008	2009	2010	2011	2012	2013	2014	2015	2016	2017	2018	2019	2020	2021	2022
Number of dogs vaccinated in this year	0	4,199	13,126	7,675	7,433	11,562	14,016	14,098	11,622	8,252	12,532	16,229	19,288	21,150	22,724	26,419	11,050	19,305	14,882	6,343	17,036
Percentage of dog population vaccinated in campaigns	0	9	28	16	15	23	26	26	21	14	21	26	30	32	33	37	15	25	19	8	20
Percentage of villages where a vaccination campaign was held	0	48	90	56	50	86	97	97	62	50	65	72	86	97	95	97	50	100	99	44	92

Table S2: Coefficients for monthly village-level GLMMs for cases/dog in a village in the current month. 95% credible intervals (CrIs) in brackets. Coefficients for fixed effects where the 95% CrI does not include zero are marked *. Model 1 is presented in Fig. 3, model 2 in Supplementary Fig. S2, and models 3 and 4 in Fig. 4.

**Coefficient**	**1. Full model without power means**	**2. Without power means or prior cases/dog**	**3. Full model with power means**	**4. Power means without prior cases beyond focal village**	**5. Power means without prior cases at any scale**
Intercept	-0.28 (-1.16-0.63)	-1.27 (-2.87-0.43)	-2.08 (-3.56--0.61)	-8.67 (-10.74--6.48)	-12.21 (-14.63--9.66)
Village vaccination over last 2 months	-0.87 (-1.43--0.33)*	-1.29 (-1.9--0.68)*			
Vaccination at borders over last 2 months	-0.27 (-1.06-0.54)	-1.15 (-2.07--0.22)*			
Vaccination in non-neighbouring villages over last two months	-0.37 (-1.41-0.67)	0.33 (-0.84-1.51)			
Village susceptibility over last 2 months			0.83 (0.27-1.37)*	0.27 (-0.27-0.81)	0.36 (-0.22-0.93)
Power mean susceptibility at borders over last 2 months			0.32 (-0.53-1.18)	1.14 (0.23-2.03)*	1.72 (0.72-2.69)*
Power mean susceptibility in non-neighbouring villages over last two months			0.59 (-0.56-1.83)	5.16 (3.6-6.68)*	6.43 (4.76-8.17)*
Village ln(cases/dog) over last 2 months	0.23 (0.21-0.25)*		0.23 (0.21-0.25)*	0.29 (0.27-0.3)*	
ln(cases/dog) at borders over last 2 months	0.14 (0.11-0.16)*		0.14 (0.11-0.16)*		
ln(cases/dog) in non-neighbouring villages over last two months	0.4 (0.35-0.46)*		0.4 (0.34-0.46)*		
ln(dogs/km^2)	-0.69 (-0.95--0.44)*	-2.89 (-3.28--2.5)*	-0.67 (-0.94--0.42)*	-1.17 (-1.53--0.85)*	-1.71 (-2.11--1.31)*
Human:dog ratio	0.33 (0.19-0.46)*	0.55 (0.24-0.84)*	0.33 (0.19-0.46)*	0.33 (0.18-0.49)*	0.44 (0.22-0.67)*
Village random effect standard deviation	0.68 (0.54-0.83)	1.69 (1.39-2.05)	0.67 (0.54-0.83)	0.82 (0.64-1.04)	1.23 (1-1.51)
size	0.34 (0.3-0.37)	0.16 (0.15-0.18)	0.34 (0.3-0.37)	0.29 (0.26-0.32)	0.16 (0.15-0.18)
p (power used in calculating power means)			2.56 (-1.26-6.19)	10.54 (8.07-13.22)	12.11 (9.64-14.67)

Table S3: Coefficients for monthly district-level GLMs for cases/dog in the district in the current month. 95% credible intervals (CrIs) in brackets. Coefficients for fixed effects where the 95% CrI does not include zero are marked *. Predictions from model 1 (and coefficients for model 2) are presented in Fig. S5). Parameters from models 3 and 4 are illustrated in Fig. S6.

**Parameter**	**1. Full model without power mean**	**2. Without power mean or prior cases/dog**	**3. Full model with power mean**	**4. Power mean without prior cases/dog**
Intercept	1.52 (0-3.1)	6.67 (4.03-9.31)	0.5 (-2.15-2.99)	8.01 (0.17-11.42)
Arithmetic mean vaccination over last 2 months	-0.75 (-2.06-0.55)	-0.06 (-2.18-2.13)		
Power mean susceptibility over last 2 months			0.92 (-0.5-2.46)	-1.32 (-2.98-4.19)
Log cases/dog over last 2 months	0.78 (0.7-0.86)*		0.78 (0.7-0.86)*	
Log dogs/km^2	-0.98 (-1.59--0.38)*	-4.71 (-5.59--3.81)*	-0.94 (-1.54--0.35)*	-4.89 (-5.72--3.68)*
size	4.76 (3.55-6.32)	1.3 (1.06-1.57)	4.79 (3.58-6.31)	1.36 (1.11-1.65)
p (power used in calculating power means)			2.02 (-1.74-5.73)	-3.06 (-7.32-6.32)

Table S4: Coefficients for the annual village-level negative binomial GLMMs. Each row represents a different model with a different combination of explanatory variables. 95% credible intervals in brackets. Coefficients for fixed effects where the 95% CrI does not include zero are marked *. Predictions from the full model (final row; including vaccination and cases/dog in the last year as explanatory variables) are presented in supplementary Fig. S7.

**Intercept**	**Mean Vaccination coverage**									**ln(cases/dog) last year**			**ln(Dogs /km²)**	**Human: Dog Ratio**	**Standard deviation of village random effect**	**Size**
	**Last year**			**Mean last 2 years**			**Mean last 3 years**			**Village**	**Borders**	**Non-bordering**				
	**Village**	**Borders**	**Non-bordering**	**Village**	**Borders**	**Non-bordering**	**Village**	**Borders**	**Non-bordering**							
1.37 (-0.96 - 3.98)	-1.12 (-1.8 - -0.43)*	-0.44 (-1.54 - 0.65)	-0.81 (-2.35 - 0.66)										-2.9 (-3.67 - -2.2)*	0.53 (0.22 - 0.85)*	1.69 (1.28 - 2.16)	0.43 (0.38 - 0.49)
-0.02 (-2.35 - 2.73)				-1.32 (-2.25 - -0.34)*	-1.38 (-2.92 - 0.15)	-1 (-3.08 - 1.08)							-2.44 (-3.29 - -1.68)*	0.56 (0.28 - 0.87)*	1.5 (1.11 - 1.97)	0.41 (0.36 - 0.47)
-0.62 (-2.97 - 2.17)							-1.48 (-2.63 - -0.29)*	-1.65 (-3.51 - 0.2)	-0.42 (-3.28 - 2.45)				-2.28 (-3.22 - -1.52)*	0.58 (0.28 - 0.87)*	1.44 (1.05 - 1.94)	0.38 (0.33 - 0.44)
1.5 (-0.03 - 3.13)	-1.07 (-1.73 - -0.38)*	-0.5 (-1.56 - 0.56)	-2.73 (-4.09 - -1.37)*							0.08 (0.02 - 0.14)*	0.16 (0.07 - 0.26)*	0.43 (0.29 - 0.56)*	-1.37 (-1.9 - -0.92)*	0.38 (0.18 - 0.58)*	1 (0.76 - 1.3)	0.49 (0.42 - 0.55)

T**able S5: Coefficients for the annual district-level negative binomial GLMs.** Each row represents a different model with a different combination of explanatory variables. 95% credible intervals in brackets. Coefficients for fixed effects where the 95% CrI does not include zero are marked *. Predictions from the full model (final row; including vaccination and cases/dog in the last year as explanatory variables) are presented in supplementary Fig. S8.

**Intercept**	**Mean vaccination coverage**			**ln(cases/dog) last year**	**ln(Dogs /km2)**	**Shape**
	**Last year**	**Mean last 2 years**	**Mean last 3 years**			
12.29 (4.84-19.74)	0.55 (-4.1-4.93)				-5.7 (-8.04--3.29)*	2.08 (1.03-3.6)
13.8 (5.6-22.2)		0.6 (-5.75-6.87)			-6.15 (-8.79--3.45)*	2.08 (0.99-3.59)
14.64 (5.61-23.25)			1.92 (-6.96-10.67)		-6.5 (-9.16--3.6)*	2.03 (0.94-3.56)
9.51 (4-15.09)	-1.08 (-4.66-2.42)			0.55 (0.21-0.89)*	-3.72 (-5.84--1.64)*	3.25 (1.5-5.81)

Table S6: Fitted distributions for incubation period (units=days), serial interval (units=days) and distance kernel (units=metres). For each epidemiological variable calculated from the contact tracing data with sample size n, we fitted gamma, lognormal and Weibull distributions. Estimates of the parameters for each distribution are provided. The best fitting model for each epidemiological variable based on AIC is highlighted in bold. The 95th, 97.5th and 99th percentiles of each distribution are given.

	n	Distribution	Parameter 1: Name	Parameter 1: Value (95%CI)	Parameter 2: Name	Parameter 2: Value (95%CI)	AIC	95th percentile	97.5th percentile	99th percentile
**Incubation Period**	1,212	Gamma	shape	1.18 (1.1-1.26)	rate	0.04 (0.04-0.05)	10421	77.2	93.9	115.9
		**Lognormal**	**meanlog**	**2.82 (2.77-2.87)**	**sdlog**	**0.95 (0.91-0.99)**	**10159**	**80.1**	**108.0**	**152.9**
		Weibull	shape	1 (0.96-1.05)	scale	27.27 (25.78-28.89)	10440	81.6	100.5	125.4
**Serial Interval**	1,156	Gamma	shape	1.18 (1.1-1.27)	rate	0.04 (0.04-0.05)	10022	79.9	97.2	119.9
		**Lognormal**	**meanlog**	**2.86 (2.81-2.92)**	**sdlog**	**0.95 (0.91-0.99)**	**9781**	**83.4**	**112.5**	**159.3**
		Weibull	shape	1 (0.96-1.05)	scale	28.29 (26.73-30.09)	10041	84.5	104.0	129.8
**Distance kernel**	6,897	Gamma	shape	0.58 (0.55-0.6)	rate	0.00038 (0.00035-0.00041)	45177	5596.7	7216.4	9407.9
		Lognormal	meanlog	6.45 (6.4-6.49)	sdlog	1.74 (1.69-1.8)	45321	11129.8	19284.6	36540.9
		**Weibull**	**shape**	**0.69 (0.67-0.71)**	**scale**	**1253.12 (1192.2-1315.93)**	**45143**	**6142.8**	**8304.8**	**11453.2**
